# Adapter dimer contamination in sRNA‐sequencing datasets predicts sequencing failure and batch effects and hampers extracellular vesicle‐sRNA analysis

**DOI:** 10.1002/jex2.91

**Published:** 2023-06-11

**Authors:** Joaquín J. Maqueda, Alberta Giovanazzi, Ana Mafalda Rocha, Sara Rocha, Isabel Silva, Nadine Saraiva, Nuno Bonito, Joana Carvalho, Luis Maia, Marca H. M. Wauben, Carla Oliveira

**Affiliations:** ^1^ BIOINF2BIO, LDA Porto Portugal; ^2^ i3S – Instituto de Investigação e Inovação em Saúde Universidade do Porto Porto Portugal; ^3^ Ipatimup – Institute of Molecular Pathology and Immunology of the University of Porto Porto Portugal; ^4^ Department of Biomolecular Health Sciences Faculty of Veterinary Medicine Utrecht University Utrecht The Netherlands; ^5^ IBMC ‐ Instituto de Biologia Molecular e Celular University of Porto Porto Portugal; ^6^ IPOC – Instituto Português de Oncologia Francisco Gentil Coimbra Portugal; ^7^ ICBAS‐UP ‐ Instituto de Ciências Biomédicas Abel Salazar University of Porto Porto Portugal; ^8^ CHUPorto – Department of Neurology Centro Hospitalar Universitário do Porto Porto Portugal; ^9^ FMUP – Faculty of Medicine University of Porto Porto Portugal

**Keywords:** adapter dimers, batch effects, extracellular vesicles, library preparation, quality control, small RNA, small RNA sequencing

## Abstract

Small RNA (sRNA) profiling of Extracellular Vesicles (EVs) by Next‐Generation Sequencing (NGS) often delivers poor outcomes, independently of reagents, platforms or pipelines used, which contributes to poor reproducibility of studies. Here we analysed pre/post‐sequencing quality controls (QC) to predict issues potentially biasing biological sRNA‐sequencing results from purified human milk EVs, human and mouse EV‐enriched plasma and human paraffin‐embedded tissues. Although different RNA isolation protocols and NGS platforms were used in these experiments, all datasets had samples characterized by a marked removal of reads after pre‐processing. The extent of read loss between individual samples within a dataset did not correlate with isolated RNA quantity or sequenced base quality. Rather, cDNA electropherograms revealed the presence of a constant peak whose intensity correlated with the degree of read loss and, remarkably, with the percentage of adapter dimers, which were found to be overrepresented sequences in high read‐loss samples. The analysis through a QC pipeline, which allowed us to monitor quality parameters in a step‐by‐step manner, provided compelling evidence that adapter dimer contamination was the main factor causing batch effects. We concluded this study by summarising peer‐reviewed published workflows that perform consistently well in avoiding adapter dimer contamination towards a greater likelihood of sequencing success.

## 1 INTRODUCTION

Extracellular vesicles (EVs) comprise a heterogeneous group of lipid bilayer‐delimited particles, which are released by cells into the extracellular space (Mateescu et al., 2017) and have been identified in many body fluids, such as blood, milk, saliva, urine, tears, etc. (Foster et al., 2016). The number of publications in the EV‐field has exponentially increased over the past 15 years due to the discovery that they could be more than only waste carriers (Van Niel et al., 2018). In fact, it is known that EVs act as vehicles in cell‐to‐cell communication by transporting intracellular material and sending messages from one cell to another (Mir & Goettsch, 2020), thus playing an essential role in the regulation of homeostasis and disease onset and progression (Hill et al., 2013). The exploration of EV cargo is leading to advances in disease biomarker discovery and therapeutics, which nurture scientists’ interest in expanding EV research for clinical applications (Xu et al., 2020). However, isolation, purification, identification and characterization of EVs and their cargoes are not always performed according to the guidelines of the International Society of Extracellular Vesicles (Théry et al., 2018; Witwer et al., 2013; Witwer et al., 2021) and the best clinical standards, which create confounding variables in downstream analysis (Giraldez et al., 2018; Mateescu et al., 2017).

Next‐generation sequencing (NGS) techniques have eased the examination of RNA derived from EVs and the study of their possible effects in recipient cells (Hill et al., 2013). In particular, sRNA has been identified in EVs from many organisms (Zhao et al., 2020) and proven to be involved in regulating gene transcription and translation in recipient cells (Liu et al., 2019; Yu & Wang, 2019). Nevertheless, the quality and quantity of the sequenced sRNA are often suboptimal due to the lack of purity of EV samples, low amount of EV‐associated sRNA and artefacts arising from EV isolation, RNA extraction, library preparation and/or sequencing (Giraldez et al., 2018; Srinivasan et al., 2019; Srinivasan et al., 2019; Van Deun et al., 2014). These observations make EV‐derived NGS data biased and often untrustworthy. Hence, several published works have detailed the steps of (s)RNA sequencing which lead to bias, and have described methods for tackling them, either in vitro or in silico (Conesa et al., 2016; Das et al., 2019; Fuchs et al., 2015; Mateescu et al., 2017; Ozsolak & Milos, 2011). However, clear and transparent technical information, mandatory to understand and replicate EV‐sRNA sequencing experiments, is often missing in publications, resulting in the lack of reproducibility across sequencing studies performed with similar materials or research questions (Simoneau et al., 2021).

**FIGURE 1:**
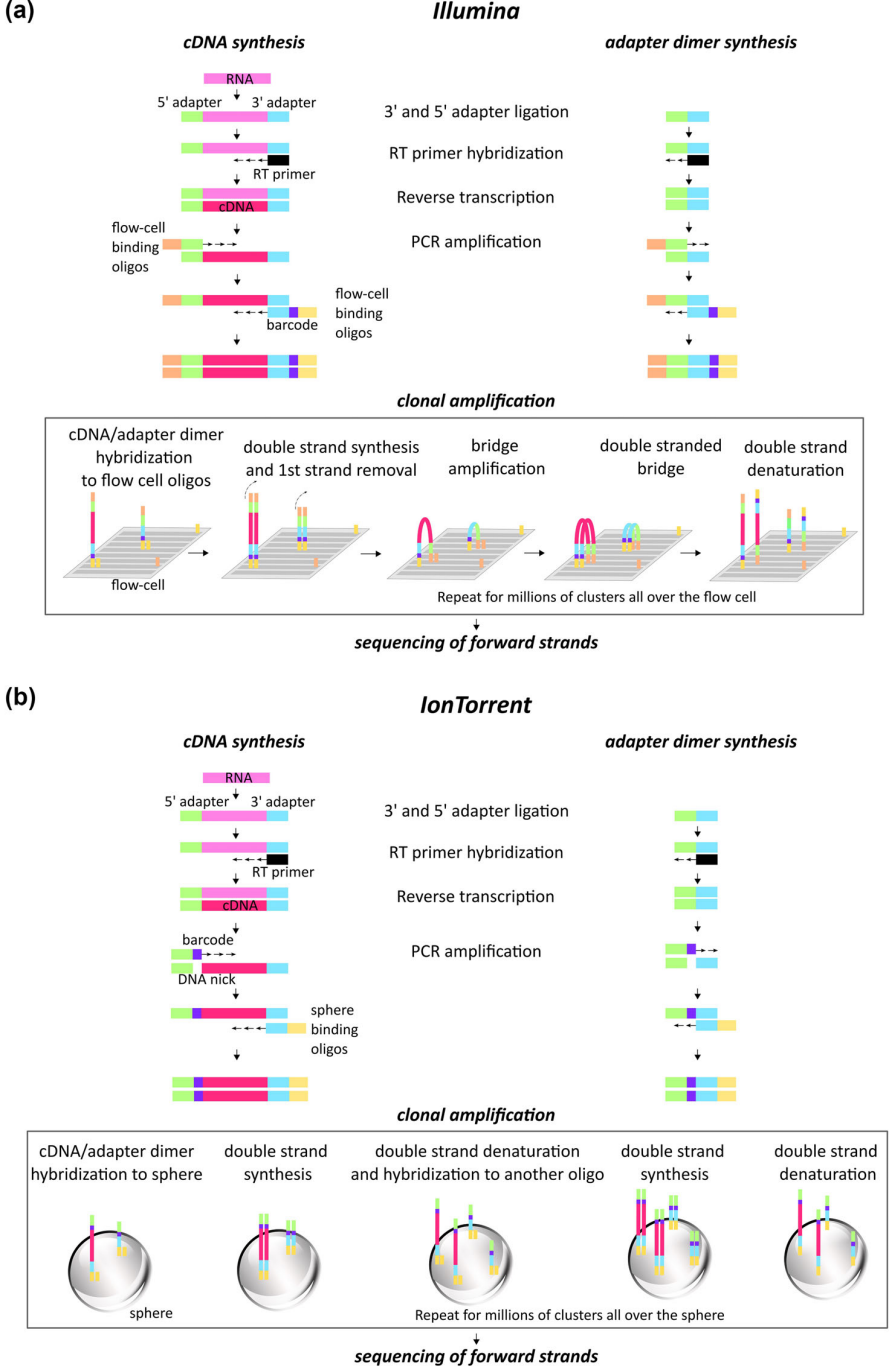
Schematic workflow of library preparation for small RNA sequencing: cDNA insert synthesis versus adapter dimer synthesis. (a) Example of Illumina workflow. After sRNA extraction, single‐strand RNA sequences (adapters) are bound to the 3’ end (3’ adapter, blue) of the sRNA molecules (pink). Then or concomitant with the 3’ adapter ligation, 5’ RNA adapters (green) are added and with the help of DNA polymerase‐reverse transcriptase (Pol/RT) and RT primers (black) cDNA synthesis occurs. Through the PCR amplification process additional motifs are introduced, that is, specific barcodes for each sample (purple) and oligos (orange, yellow) which will bind the flow cell for clonal amplification and sequencing. Adapter dimers are formed when 5’ adapters ligate to 3’ adapters with no insert (sRNA, pink) in between. They contain the full sequence of both adapters and are able to bind the flow cell and be sequenced. Hybridization to the flow cell is enabled by the flow‐cell binding oligos. Original strands are washed away once the template sequences are polymerized. Then, the bridge amplification occurs for millions of clusters all over the flow cell, thus enabling the massive replication of the template sequences. The same process happens for adapter dimer sequences, which cluster even more efficiently. Reverse sequences are washed away so that only forward template sequences go through the sequencing‐by‐synthesis process. (b) Example of Ion Torrent workflow. Library preparation is developed similarly to (a). However, barcodes are added to the 5’ end of the insert cDNA. Then, hybridization to the sphere is enabled by the 3’ sphere binding oligos. Then, the emulsion PCR occurs and the same sequences are replicated for millions of times in each sphere. Adapter dimers are able to follow the same process. Template sequences are kept so that sequencing corresponds to the original forward ones.

A common issue with EV‐sRNA NGS data is adapter dimer contamination, which originates in the library preparation step (Sheng et al., 2017). Library preparation is the process of adding artificial sequences of nucleotides (single‐stranded 5’ and 3’ adapters) to RNA for its amplification and sequencing (Figure 1, ‘cDNA synthesis’). Briefly, an adapter is bound to the 3’ end of the sRNA molecule. Subsequently, or concomitantly with the 3’ adapter ligation, 5’ adapter is added, and with the help of DNA polymerase‐reverse transcriptase (Pol/RT) and RT primers, cDNA synthesis occurs. The cDNA‐adapter sequences are then substrates for PCR amplification. Additional adapter sequences (flow‐cell/sphere‐binding oligos) are added to these cDNA constructs for hybridization of the cDNA strands to the flow cell (*Illumina*) or Ion Sphere Particles (*Ion Torrent*), which are needed for clonal amplification and sequencing (only examples of *Illumina* and *Ion Torrent* sRNA sequencing techniques are herein addressed) (Dard‐Dascot et al., 2018; Shore et al., 2016).

Adapter dimers are formed when 5’ adapters ligate to 3’ adapters with no RNA insert in between (Shore et al., 2018). They contain the full sequence of both adapters and are able to bind to the flow cell/sphere and be sequenced (Figure 1) (Bourlat et al., 2016). Furthermore, due to their smaller size, adapter dimers amplify and cluster more efficiently than the library of interest (Head et al., 2014). Contamination of the library with adapter dimers may waste the capacity of the flow cell/sphere (Quail et al., 2008). As a consequence, reads corresponding to low expressed genes often dwindle to nothing, resulting in false negative data (Shore et al., 2016). Numerous factors can give rise to adapter dimers; namely, low quality/quantity of starting material, excess of incorporated adapter sequences, imprecise size‐selection and inefficient bead clean‐up (Illumina 2020).

The amount of RNA, the presence of different RNA biotypes, as well as the RNA distribution in different EV subsets from biological sources is generally unknown (Kim et al., 2017). It is also unknown if RNA is present in all EVs, and whether different EV populations differ in their RNA cargo (de Voogt et al., 2021). Additionally, limited biological material (e.g., clinical samples or samples from small experimental animals), suboptimal biological sample collection and/or storage and different methods of EV isolation may return diverse sRNA yield, sometimes poor RNA integrity (degraded RNA) and low RNA purity (presence of contaminants) (Belder et al., 2016; Kumar et al., 2020; Shore et al., 2016; Sódar et al., 2016; Zonneveld et al., 2014). Monitoring the likelihood of success in sequencing this type of material, prior to library construction is necessary. The feasibility of preventing adapter dimer contamination has been proven through several adjustments during the library preparation process (pre‐sequencing modifications). However, a considerable amount of EV‐sRNA related works have disregarded this step. This urges the evaluation of adapter dimer contamination after library construction to predict the success of a given sequencing experiment.

Related or not with adapter dimer contamination, one of the most overlooked Quality Control elements for sequencing data are batch effects (Sheng et al., 2017). Batch effects are differences between sets of samples caused by technical variables (Bin et al., 2017), such as library preparation performed in different days, sequencing samples in two rounds or in different facilities (Leek et al., 2012). Inter‐sample differences in RNA quality and quantity are also major sources of batch effects in transcriptomics datasets (Fasold & Binder, 2014). If uncorrected, batch effects may have an unfavourable and misleading impact on downstream analysis (Zhang et al., 2020).

Although widely recognized as independent limiting factors in EV‐sRNA NGS experiments, adapter dimer contamination has not been reported as source of batch effects. The sequencing of adapter dimers hinders the sequencing of the biological cDNA, in such a way that can affect the correlation of sRNA read abundance with the original sRNA content in EVs. Moreover, if the amount of adapter dimers differs between samples, a batch effect may be generated, thus impairing the consistency of replicates and experimental conditions.

In this paper, we used outputs of pre‐sequencing quality control steps of four independent experiments to predict sequencing outcomes biasing biological results. In addition, we reviewed the literature to explore the main pre‐sequencing modifications performed in published studies to validate our findings. Finally, we provide possible solutions and a quality control pipeline for improving and predicting sRNA sequencing outcomes.

## 2 MATERIALS AND METHODS

### 2.1 EV isolation and characterization from human milk

Human milk for EV isolation was collected during routine hospital check‐ups from 20 non‐allergic mothers and 20 allergic mothers enrolled in the ACCESS study (Comparison of Human Milk Extracellular Vesicles in Allergic and Non‐allergic Mothers, NL 47426.099.14; RTPO 914; approved by the Martini Hospital Medical Ethics Committee (Groningen, the Netherlands) in accordance with the local ethical guidelines and the Declaration of Helsinki for medical research involving human subjects). Informed consent to be included in the ACCESS study was obtained from all donors. Donors were lactating women aged 18–37 years between 4 and 13 weeks after vaginal delivery of a full‐term newborn. Allergic donors were characterized by total serum IgE ≥ 50 kU/L and/or specific IgE for grass pollen, tree pollen, house dust mite, cat dander, or dog dander ≥0.35 kU/L (Phadiatop assay Thermo Scientific, Uppsala, Sweden). Non‐allergic donors had IgE < 50 kU/mL and Phadiatop antigen specific IgE < 0.35. Milk EVs were purified using a validated protocol for human milk EV isolation (Van Herwijnen et al., 2016; Zonneveld et al., 2014). Shortly, milk was prevented from cooling down and within 20 min after collection, centrifuged twice at 3000 × *g* for 10 min at 23°C (Beckman Coulter Allegra X‐12R, Fullerton, CA, USA). The cell and fat free milk supernatant was stored at −80°C until further processing. Stored milk supernatant was subsequently centrifuged for 30min at 4°C at 5000 × *g* and 10,000 × *g* (Beckman Coulter Optima L‐90K with a SW40Ti rotor). Next, 3.5–4.0 mL aliquots of the 10,000 × *g* supernatant were loaded on top of a 2.5M–0.4 M sucrose gradient (SW40 tube). Gradients fractions of 500 μL were collected and fractions containing EVs (densities 1.12–1.22 g/mL) were pooled, diluted with PBS (Invitrogen) and pelleted at 192,000 × *g* for 65 min at 4°C (Beckman Coulter Optima L‐90K with a SW40Ti rotor). Pellets were resuspended in 700 μL Qiazol (Qiagen, Hilden Germany) and frozen at −80°C until RNA extraction for sequencing. Particle concentration and size of milk EV samples of allergic and non‐allergic milk donors has been determined by Nanoparticle Tracking Analysis (NTA) in a previous study (Giovanazzi et al., 2022).

### 2.2 RNA extraction from human milk EVs

RNA enriched in microRNA (miRNA) was isolated using the miRNeasy micro kit (Qiagen, Hilden, Germany) according to manufacturer's instructions. Quality and quantity of the RNA samples were assessed using Agilent 2100 Bioanalyzer pico‐RNA chips (Agilent Technologies Netherlands B.V., Amstelveen, the Netherlands) and the concentration of isolated small RNA (25‐200nt) varied between 0.9–26 ng/μL for individual milk EV donors.

### 2.3 Small RNA library preparation and sequencing from human milk EV‐RNA

The Illumina Truseq small RNA Sample Prep Kit was used to process the samples according to the kit‐specific guidelines. Size fractions containing inserts of 22–30 nt in length were excised. The quality and yield after sample preparation was measured with the Fragment Analyzer (Advanced Analytical Technologies, Inc.). 1.6 pM cDNA library was used for sequencing. sRNA sequencing was done on the Illumina NextSeq 500 platform with 75 bp single‐end sequencing. Two flow cells were used in order to obtain a minimum of 7 million sequencing reads per sample.

### 2.4 Pre‐processing of human milk EV‐sRNA sequencing data

For each individual sample, four lanes were combined in a single fastq file. Subsequently, we combined the files for those samples which were sequenced in two different flow cells. 40 fastq files were obtained. FastQC (version 0.11.5) (Andrews et al., 2015) was used to perform quality control checks on sequencing data. TruSeq small RNA 3’ adapter sequences were checked and clipped from all reads using cutadapt (version 2.8, with settings of “‐a TGGAATTCTCGGGTGCCAAGGAACTCCAGTCAC –error‐rate 0.1 –times 1 ‐m 15”). Remaining primer sequences were removed using cutadapt (version 2.8, with settings of “‐g CGACAGGTTCAGAGTTCTACAGTCCGACGATC –error‐rate 0.1 –times 1 ‐m 15”) (Martin, 2011). Low quality bases (*q* < 20) were trimmed from all sequence reads using sickle (version 1.33, with settings of “‐t sanger ‐l 15”) (Joshi & Fass, 2011). Finally, all reads shorter than 15 bases were discarded. Here, cutadapt was also used to estimate the percentage of adapter dimers in each sample.

### 2.5 Data analysis of human milk EV‐sRNA sequencing

Processed reads were aligned to human reference genome GRCh38 from Ensembl (Homo sapiens primary assembly, soft‐masked) (Cunningham et al., 2015). Number of aligned reads per each annotated sRNA was calculated. These processes were performed by Manatee algorithm (version 1.2) (Handzlik et al., 2020). Bowtie was used to align reads (version 1.0.1, with settings of “–best –strata ‐m 50 ‐k 3 ‐v 3”) (Langmead et al., 2009). Alignments to a maximum of 50 loci were allowed and up to 3 of them were reported, with a maximum of 3 mismatches. Manatee counts output was rounded and used as raw expression data. The sRNA annotation track was obtained from miRBase (version 22.1) (Kozomara & Griffiths‐Jones, 2014), GtRNAdb (version 2.0, January 2016) (Chan & Lowe, 2016) and Ensembl 99 (Cunningham et al., 2015) in order to analyse the following biotypes: miRNA, tRNA, misc_RNA, Mt_tRNA, ribozyme, rRNA, scaRNA, scRNA, Mt_rRNA, snoRNA, snRNA and vaultRNA. Sample distribution representing miRNA‐levels (counts‐per‐million in log_2_) was depicted in a MDS plot by using edgeR package (Robinson et al., 2009).

### 2.6 Blood sample collection from gastric cancer (GC) patients

Patients with Gastric Cancer (GC) enrolled in this study were admitted at Instituto Português de Oncologia Francisco Gentil (Coimbra, Portugal) for regular haemogram analysis. Peripheral blood samples (5 mL) were collected from four GC patients at three different timepoints (12 samples in total), between April 2015 and May 2017. Samples were collected into room temperature spray‐dried K2EDTA anticoagulant tubes (BD Vacutainer PPT Plasma Preparation Tube) and processed into plasma following manufacturer's instructions. Briefly, within 30 min after collection, the PPT tubes containing whole blood were gently inverted 8–10 times and centrifuged at room temperature (18–25°C) for a minimum of 10 min at 1100 × *g*. Plasma (1.5–3 mL) was decanted into a new tube and samples were stored at −80°C until further processing. The study was approved by the hospital's ethics committee (ref. Ipatimup/IPO de Coimbra_2014), and written informed consent was obtained from all patients before sample collection, in accordance with the Declaration of Helsinki on medical research involving human subjects.

### 2.7 EV enrichment and characterization from human GC plasma samples

Thawed plasma samples (1.5–3 mL) were diluted with 0.9% NaCl (pH 7.4) to a final volume of 15 mL and filtered through a 0.22 μm filter. Filtered supernatants were centrifuged in a SW32 rotor (Beckman Coulter, Fullerton, CA, USA) at 100,000 × *g*, for 14 h at 4°C to pellet EVs. EV‐enriched pellets were washed in 0.9% NaCl (pH 7.4), centrifuged at 100,000 × *g* for 2 h at 4°C, resuspended in a volume of 0.9% NaCl and stored at 4°C. Particle size and concentration were determined by NTA using the NanoSight NS300 instrument (Malvern, Worcestershire, UK) with scientific CMOS camera. For this, EV‐enriched samples were diluted (1:500) in 0.9% NaCl. Three technical measurements were recorded under a controlled fluid flow with a pump speed set to 40 and a camera focus level adjusted between 10 and 16. The three videos were processed using the NTA 3.1 Build 3.1.54 software. Particle concentration and mean size of EVs are reported in Figure S1a.

### 2.8 RNA extraction from EV‐enriched human GC plasma samples

Prior to RNA isolation, EV‐enriched samples were incubated with RNAse A at 37°C for 10 min (final concentration 0.4 mg/mL; NZYTech, Lisbon, Portugal). RNAse A was inhibited with RNasin ribonuclease inhibitor (final concentration 1 U/μL; Promega, Madison, WI, USA). Next, EV‐enriched samples were treated with proteinase K at 37°C for 10 min (final concentration 0.05 mg/mL; Qiagen, Hilden, Germany), which was inactivated at 75°C for 10 min. Small RNA (sRNA) was isolated from RNAse A/Proteinase K‐treated samples with the mirCURY RNA isolation kit ‐ Biofluids (Exiqon, Vedbaek, Denmark), according to manufacturer's instructions. Concentration and quality of sRNA, including miRNA, were measured using the Agilent 2100 Bioanalyzer with the small RNA kit (Agilent, Santa Clara, CA, USA). The concentration of miRNA (10–40 nt) was in the range of 0.3–1.7 ng/μL. Purified sRNA samples were kept at −80°C until further analysis.

### 2.9 Small RNA library preparation and sequencing from EV‐enriched human GC plasma ‐sRNA

Isolated sRNA was used for small RNA library preparation using the Ion Total RNA‐Seq Kit v2 (Thermo Fisher Scientific, Waltham, MA, USA), according to manufacturer's instructions. Briefly, 3′ and 5′ adapters were attached directionally, and simultaneously, to 3 μL of sRNA input (0.94–5.23 ng miRNA). Hybridized and ligated RNA was reversed transcribed using Ion RT primer v2 and SuperScript III Enzyme mix. Each cDNA sample was amplified and barcoded using Platinum PCR SuperMix High Fidelity, Ion Xpress RNA 3′ Barcode Primer, and a unique Ion Xpress RNA‐Seq Barcode BC Primer, which allows sample identification and tracking. Size distribution of amplified cDNA library was measured using the Agilent 2100 Bioanalyzer with Agilent DNA 1000 Kit (Agilent, Santa Clara, CA, USA). Due to the high amount of adapter dimers, an adapted protocol was implemented with the aim of reducing the quantity of adapter dimers and enriching the library of interest. Library selection and sequencing test run were performed. The first approach consisted of size selection with E‐Gel SizeSelect 2% Agarose Gel (Thermo Fisher Scientific), which decreased the proportion of adapter dimers. For less concentrated libraries size selection was performed by using 4% Agarose Gel cut, and purification of the band corresponding to ≈110 bp sequences was performed with illustra GFX PCR DNA and Gel Band Purification Kit (GE Healthcare, New York, USA). Agilent 2200 TapeStation (S/N 3‐PM‐1173NA)—HS D1000 Screen Tape (P/N 5067–5584) was used for size distribution control. An equal volume (3 μL) of each library was used to prepare the final pool. Pooled libraries were processed on Ion Chef System (S/N CHEF00657) using the Ion 540 Kit‐Chef (P/N A27759) and the resulting 540 chip (P/N A27766) was sequenced on Ion S5 XL System (S/N 245717100156). Fastq files were generated using the Torrent Suit plugin FileExporter v5.0.

### 2.10 Pre‐processing of EV‐enriched human GC plasma sRNA sequencing data

We obtained 12 fastq files. FastQC (version 0.11.5) (Andrews et al., 2015) was used to perform quality control checks on sequencing data. Remaining Ion Torrent sRNA adapter sequences were checked and clipped from all reads using cutadapt (version 2.8, with settings of “‐b ATCACCGACTGCCCATAGAGAGGAAAGCGG –error‐rate 0.2 –times 1 ‐m 15 ‐q 20”) (Martin, 2011). Low quality bases (*q* < 20) were trimmed from 3’ end of the reads. Finally, all sequence reads shorter than 15 bases were removed. Cutadapt was also used to estimate the percentage of adapter dimers in each sample.

### 2.11 Data analysis of EV‐enriched human GC plasma sRNA sequencing

Data analysis was performed as previously described in ‛Data analysis of human milk EV‐sRNA sequencing’.

### 2.12 RNA extraction from human Formalin‐Fixed Paraffin‐Embedded (FFPE) GC tissues

Formalin‐fixed paraffin‐embedded (FFPE) tumour tissues from four surgical specimens, from the GC patients (described above), were histologically analysed and showed to contain a minimum of 70% of neoplastic cells. To maximize the recovery of sRNA, slices of 20 μm in thickness (one slice per FFPE sample) were subjected to total RNA isolation using the RecoverAll Total Nucleic Acid Isolation Kit for FFPE (Ambion, Austin, TX, USA) according to the manufacturer's protocol. The concentration and quality of total RNA were measured using the Agilent 2100 Bioanalyzer with the small RNA kit (Agilent, Santa Clara, CA, USA). The concentration of miRNA (10–40nt) was in the range of 1.9–7.7 ng/μL. RNA was kept at −80°C until further analysis.

Library preparation, sequencing and data analysis for four FFPE tissue RNA samples was performed as described in ‛Small RNA library preparation and sequencing from EV‐enriched human GC plasma sRNA’, ‛Pre‐processing of EV‐enriched human GC plasma sRNA sequencing data’ and ‛Data analysis of human milk EV‐sRNA sequencing’, respectively.

### 2.13 Mice information and plasma sample collection

Wild‐type C57BL6 mice were anaesthetized and blood was obtained from the inferior vena cava using EDTA‐coated syringes and centrifuged at 2000 × *g* at RT for 10 min to obtain plasma. Plasma samples were aliquoted into 1.5 mL low‐bind Eppendorfs and stored at −80°C, until further use. Needle to freezer time was below 30 min. A total of 16 samples were analysed. This study was approved by the Portuguese National Regulatory Agency for animal studies (DGAV approval n° 003576).

### 2.14 EV‐enrichment and characterization from mouse plasma

Mouse plasma EV‐enriched samples were obtained by differential centrifugation. Once thawed, plasma samples were transferred to a clean tube and diluted with a Sodium Chloride (NaCl) 0.9% solution (pH 7.4) to a final volume of 13 mL and filtered using a 0.22 μm filter. Filtered plasma was ultracentrifuged in a SW32 rotor (Beckman Coulter, Fullerton, CA, USA) at 100,000 × *g* overnight at 4°C to pellet EVs. EV‐enriched pellets were resuspended with NaCl and ultracentrifuged at 100,000 × *g* for 2 h at 4°C. The supernatant was discarded and pellets were resuspended in an appropriate NaCl 0.9% solution volume and stored −80°C. EV‐enriched pellets were evaluated for particle size and concentration by NTA (Figure S1b), as described above in ‛EV enrichment and characterization from plasma of GC patients’.

### 2.15 RNA extraction from EV‐enriched mouse plasma samples

RNA was isolated from EV‐enriched preparations using the miRNeasy Serum/Plasma Kit (Qiagen) according to manufacturer's instructions. Concentration and quality of sRNA, including miRNA, were measured using the Agilent 2100 Bioanalyzer with the sRNA assay (Agilent, Santa Clara, CA, USA). The concentration of miRNA (10–40 nt) was in the range of 0.4–4.3 ng/μL.

### 2.16 Small RNA library preparation and sequencing from mouse plasma EV‐enriched sRNA

Isolated RNA samples were used for small RNA library preparation using the protocol previously described in ‛Small RNA library preparation and sequencing from EV enriched human GC plasma sRNA’, with the exception that the protocol was not adapted to remove adapter dimer products.

### 2.17 Pre‐processing of EV‐enriched mouse plasma sequencing data

We obtained 16 fastq files. Pre‐processing was performed as described in ‛Pre‐processing of EV‐enriched human GC plasma sRNA sequencing data’.

### 2.18 Data analysis of EV‐enriched mouse plasma sRNA‐seq

Data analysis was performed as previously described in ‛Data analysis of human milk EV‐sRNA sequencing’.

### 2.19 Literature search strategy

In order to get an overall view of the different protocols used in EV‐sRNA experiments, and the pre‐sequencing modifications developed to overcome adapter dimer contamination, we collected available EV‐sRNA datasets published between 2018 and 2021. Peer‐reviewed published articles were searched with the help of Gene Expression Omnibus (GEO) (Barrett et al., 2013) DataSets using the following keywords: ‘extracellular vesicle’ or ‘extracellular vesicles’. Only studies corresponding to ‘non‐coding RNA profiling by high throughput sequencing’ were included and, among these, only articles with the following criteria were selected: clear description of materials and methods, known adapter sequences, available raw data (original SRA/fastq files, with no reads pre‐processing), a minimum of six samples per dataset, and showing solutions for prevention/correction of adapter dimer contamination. Selected datasets were analysed and FastQC (version 0.11.5) (Andrews et al., 2015) was used to perform quality control of downloaded raw data.

## 3 RESULTS

### 3.1 Featured sequencing datasets

**FIGURE 2:**
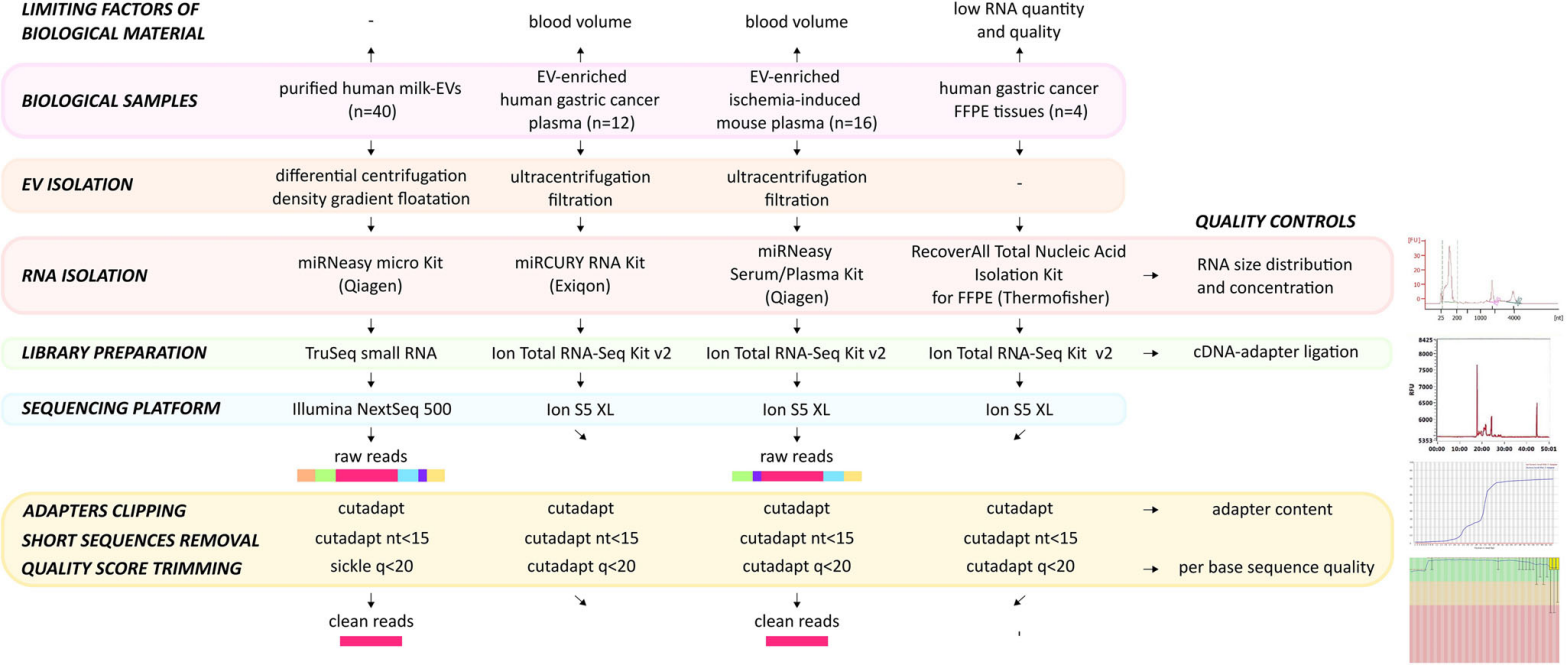
Workflow of biological sample preparation, small RNA‐seq and reads pre‐processing of the four experimental datasets analysed. The complete workflow from biological sample preparation to the pre‐processing of raw sequenced reads is depicted. One dataset comprises the sequencing of sRNA isolated from purified human milk EVs. Two datasets are derived from EV‐enriched blood plasma, from human gastric cancer patients or mice samples. The fourth sRNA‐seq dataset is derived from Formalin‐Fixed Paraffin‐Embedded (FFPE) tissues from gastric cancer patients.

We analysed sRNA sequencing data generated from four independent experiments performed in different laboratories and sequencing facilities in Portugal and the Netherlands (Figure 2). These datasets include sequencing data of RNA extracted from purified human milk EVs of healthy and allergic donors, from EV‐enriched blood plasma samples (from human GC patients and mice), and from human gastric cancer tissues.

The first experiment enclosed a sequencing dataset of EV‐RNA isolated from 40 human milk samples. The second experiment included a sRNA‐sequencing dataset obtained from 12 EV‐enriched plasma samples from four gastric cancer patients. The third experiment comprised sRNA‐sequencing data derived from EV‐enriched plasma samples from 16 mice. To further confirm that other limited biological material (low quantity/quality RNA sources) such as formalin‐fixed paraffin embedded tissues may be prone to unsuccessful NGS results, we analysed a sRNA‐sequencing dataset from 4 human surgical FFPE tissues deriving from gastric cancer patients.

Although the purpose of these experiments, the biological sample processing and the sequencing platforms used were different; the downstream bioinformatics workflow was comparable (Figure 2).

### 3.2 Analysis of raw and clean reads revealed a marked read loss after pre‐processing

**FIGURE 3:**
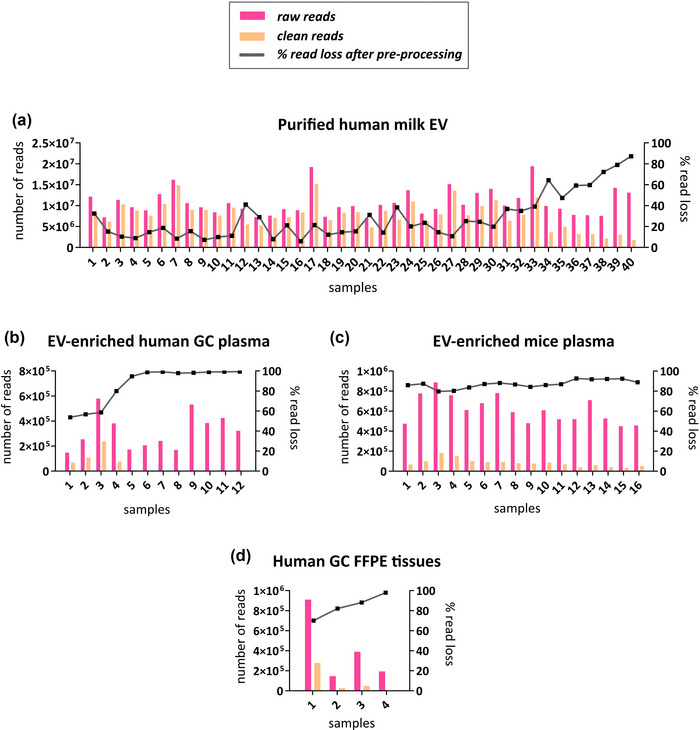
Comparison between the number of raw and clean reads. Number of raw reads (pink) and clean reads (orange) are shown (left y‐axis), which were sequenced from purified human milk EVs (a), EV‐enriched plasma samples from human gastric cancer patients (b), EV‐enriched plasma samples from mice (c) and FFPE tissue samples from gastric cancer patients (d). Percentage of read loss after pre‐processing is depicted (right y‐axis, grey line).

Pre‐processing of sRNA sequencing reads was performed on sequencing files through adapter‐clipping and quality‐trimming, and reads smaller than 15 nucleotides (nt) were removed (Figure 2). We next compared the number of reads remaining after pre‐processing (clean reads) with the total number of sequenced (raw) reads (Figure 3). To visualize the differences between raw and clean reads in individual samples, the percentage of read loss was plotted (Figure 3).

Among the 40 milk EV samples there was a 28% read loss on average. Remarkably, the average percentage of read loss in samples 34–40 was higher compared to the average of samples 1–33 (66% vs. 19%, respectively) (Figure 3a). Similarly, in the EV‐enriched GC plasma dataset differences in read loss were observed between individual samples (Figure 3b). Specifically, samples 4–12 showed an average of 97% read loss, while in samples 1–3 the average read loss was 57% (Figure 3b). In the EV‐enriched mouse plasma samples, all 16 samples had a high percentage of read loss, ranging from 80% to 92% (Figure 3c). In FFPE samples, it ranged from 70% to 97% (Figure 3d).

Overall, data presented above clearly show that after pre‐processing, there was an extremely high percentage of read loss in EV‐enriched plasma and FFPE samples, which resulted in a very low percentage of clean reads remaining after pre‐processing. Also in the purified human milk EV samples, a cohort with relatively high number of reads, substantial read loss was observed.

Given that both EV‐enriched human plasma samples and milk‐EV samples displayed marked differences between individual samples with respect to the percentage of read loss, we next focused on the differences in read loss between individual samples. We ordered samples from the lowest to the highest percentage of read loss and applied a linear regression model fitting all data points from each experiment (Figure S2). Based on the slope of the line and the optimal goodness‐of‐fit of the linear regression model (R‐squared, R^2^), two groups of samples were identified in the human milk EV experiment and three in the EV‐enriched human plasma GC experiment (Figures S2a and S2b, respectively). In contrast, only one group was identified in the EV‐enriched mouse plasma experiment and in the FFPE tissue GC samples, preventing further sample stratification in these datasets (Figure S2c and S2d, respectively). The linear regression model of the human milk EV experiment dataset showed that the slope of the fitted line in milk samples 34–40, in which substantially more read loss was observed, is noticeably steeper compared to samples 1–33 (6.242 vs. 1.058). In the EV‐enriched GC plasma samples 3, 4, 5, 8, the value of the slope was 13.240 and reached a plateau around 96% of read loss (samples 8, 9, 6, 10, 7, 11, 12), compared to 2.469 for samples 1–3.

Overall, this approach reveals two groups of samples with clearly different read loss percentages after pre‐processing, both in milk and GC plasma experiments. To understand the cause of the varied read loss, we next investigated every wet‐lab step downstream EV isolation for each dataset, as well as the available quality control outputs (Figure 2).

### 3.3 Extent of read loss between individual samples of a dataset is not correlated with isolated RNA quantity

**FIGURE 4:**
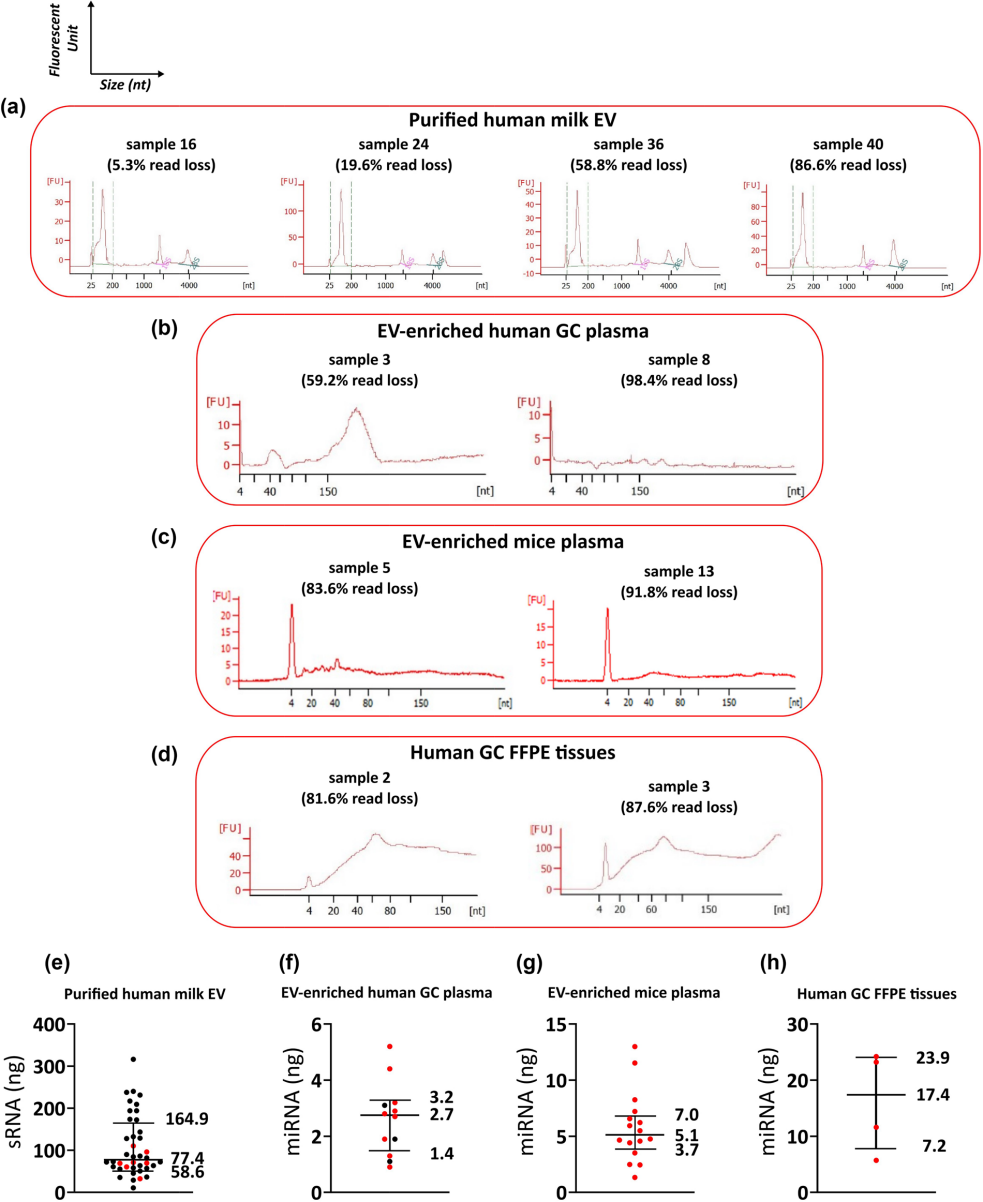
Quality control of isolated RNA. Quantity (ng) and size distribution of isolated RNA were determined by Bioanalyzer total/small RNA electropherograms. Representative electropherograms for samples with relatively low and high % read loss are shown for milk‐EVs (a), EV‐enriched plasma samples of gastric cancer patients (b), EV‐enriched mice plasma samples (c) and FFPE tissue samples from gastric cancer patients (d). Boxplots of sRNA and miRNA quantities of each individual sample are depicted for the four experiments (e, f, g, h). Median and interquartile ranges are represented and samples classified in the group of highest read loss are highlighted in red.

RNA extracted from the different biological samples was quantified and analysed for size distribution using RNA electropherograms obtained with Bioanalyzer (Figure 4). Total RNA electropherograms indicated that all 40 milk EV samples were characterized by high concentrations of sRNA (25–200 nt), regardless of the percentage of read loss (Figure 4a). sRNA quantities varied between 10.7 ng and 316.1 ng for all milk samples (median = 77.4 ng) (Figure 4e and Table S1). In the milk EV samples showing a relatively high % of read loss, only sample 34 contained a comparably lower sRNA concentration (32.5 ng), while it ranged from 60.5 to 110.3 ng in samples 35–40. These data indicate that the marked read loss in milk samples 34–40 did not correlate with the amount of sRNA used for sequencing.

The amount of miRNA (10–40 nt) isolated from plasma samples in the GC experiment was generally low (range of 0.9–5.2 ng (median = 2.7 ng)) (Figure 4b,f and Table S2). Samples characterized by the highest percentage of read loss (samples 4–12) were not different from the other samples in this dataset regarding miRNA quantity. Also in this dataset, globally characterized by a low quantity of RNA used for sequencing, we did not find an association between miRNA quantity and read loss.

Similar to the GC experiment, no correlation was found between the percentage of read loss and RNA quantity in the mice plasma EV‐enriched dataset, as only low amounts of miRNAs were available from all samples (range: 1.3–13 ng; median = 5.1 ng), and all were indistinctly characterized by a percentage of read loss between 80% and 90% (Figure 4c,g and Table S3).

In FFPE GC tissue samples, the miRNA input material range was wide (5.7–42 ng; median = 17.4 ng), however, low miRNA quantity was not correlated with massive read loss, and samples with greater read loss were also those with greater miRNA input material (Figure 4d,h and Table S4).

All 72 samples from the three datasets described above were further analysed to try to understand the cause of the marked read loss after pre‐processing.

### 3.4 cDNA electropherograms revealed a constant peak which correlated with the degree of read loss

**FIGURE 5:**
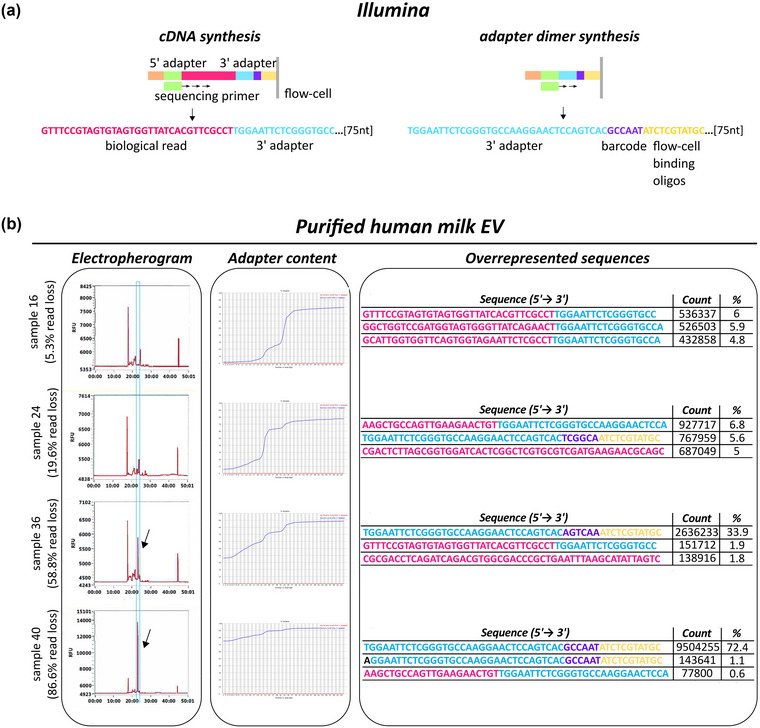
cDNA library preparation and sequencing quality controls for Illumina technology. (a) Graphical representation of Illumina sequencing technology, both for sequences with or without insert cDNA sequence. Read length was equal to 75 nucleotides. Colours match Figure 1. (b) Quality control of library preparation and sequencing of milk‐EVs (See Figure S2 for all 40 samples). The electropherogram shows a peak around 25 min, which represents the sRNA libraries and, in case there is adapter dimer contamination, one arrow indicates a peak around 23 min (blue box). Adapter content graphs and overrepresented sequences tables from FastQC are depicted in order to monitor the presence of adapter dimers. Sequencing mismatches inside adapter sequences are shown in black. Font colours match (a). Every read where the 3’ adapter has been identified in its 5’ end corresponds to an adapter dimer.

**FIGURE 6:**
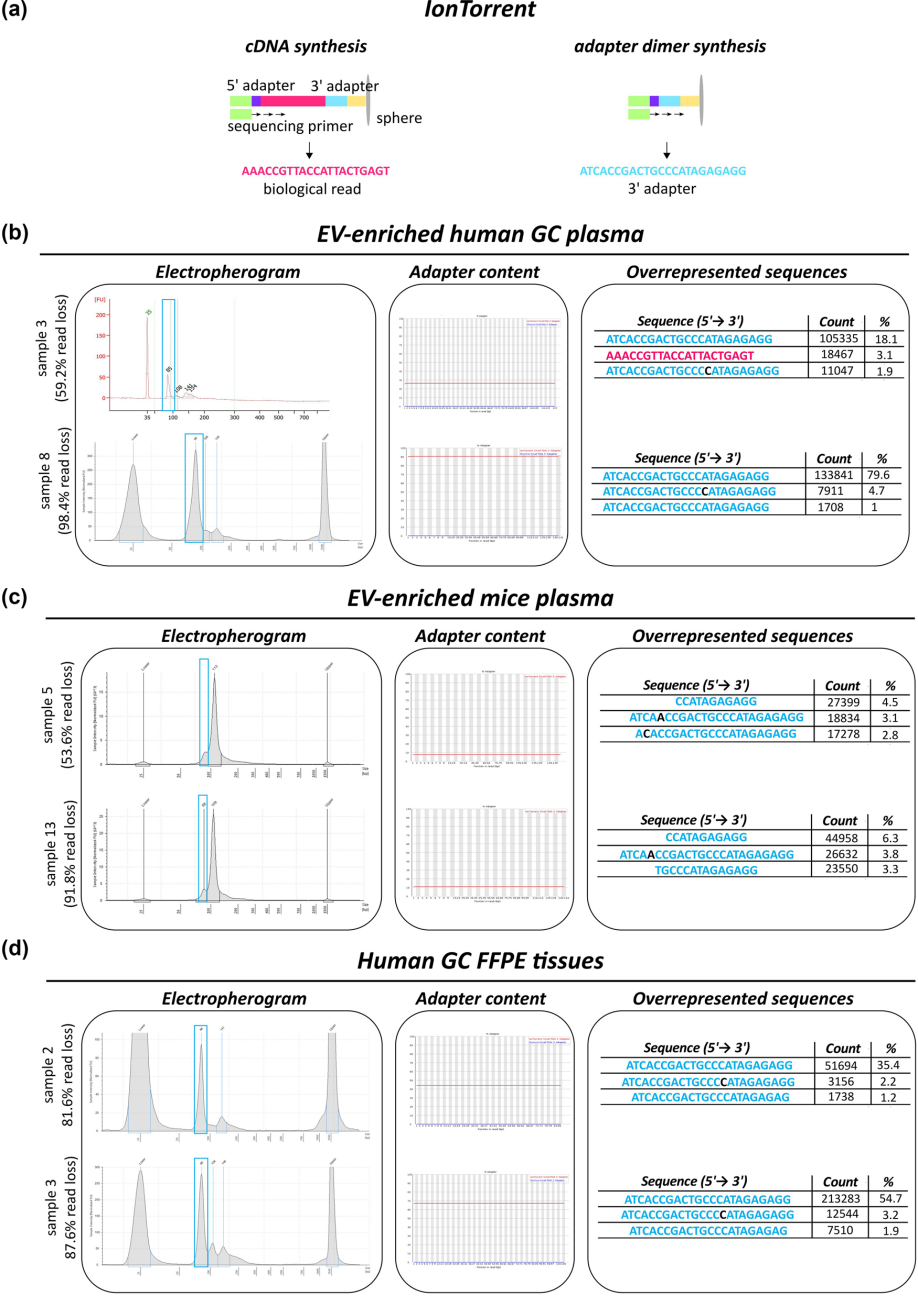
cDNA library preparation and sequencing quality controls for Ion Torrent technology. (a) Graphical representation of Ion Torrent sequencing technology, both for sequences with or without insert cDNA sequence. Colours match Figure 1. (b–d) Quality control of library preparation and sequencing of EV‐enriched GC plasma, mice and FFPE experiments (See Figures S3–S5). The electropherogram shows a peak around 110bp, which represents the sRNA libraries and, in case there is adapter dimer contamination, it shows a peak around 88 bp (blue boxes). Adapter content graphs and overrepresented sequences tables from FastQC are shown in order to monitor the presence of adapter dimers. Sequencing mismatches inside adapter sequences are shown in black. Font colours match (a). Every read where the 3’ adapter has been identified in its 5’ end corresponds to an adapter dimer.

Following RNA extraction, sRNA was used as input for the library preparation, and the quality of cDNA after library preparation was measured by Fragment Analyzer, Bioanalyzer or Tapestation softwares. For each dataset, the resulting size of cDNA sequences was plotted in electropherograms. These graphs showed, in some of the milk samples, a peak corresponding to a shorter DNA size (at approximately 23 min of migration) compared to sRNA size (Figure 5b blue box, and Figure S2). In the EV‐enriched GC human plasma and mice plasma, as well as in FFPE tissue samples, in addition to the peak around 110 bp, which corresponds to the sRNA of interest, a peak at 88bp could be detected (Figures 6b–d blue boxes, and Figures S4–S6). Particularly in the milk EV samples, but transversally to all samples in this study, the presence and height of these additional peaks correlated with the percentage of read loss (Figures 5 and 6). Of note, when assessing the quality of the library preparation via electropherograms, a peak at around 120 bp for *Illumina* (Illumina 2020) and 88 bp for *Ion Torrent* (Fisher Scientific, 2022 Jul 11) are indicative of the presence of adapter dimers.

### 3.5 The FastQC report confirmed the presence of adapter dimer contamination

In order to further investigate the presence of adapter dimers in these datasets, the FastQC report, which includes information on raw data generated by high throughput sequencing experiments, was further examined. The FastQC report contains several quality control modules, such as, among others, ‛Adapter content’, ‛Overrepresented sequences’ and ‛Per base sequence quality’, which can help to detect potential anomalies which originate in the sequencing process.

#### 3.5.1 Adapter content

FastQC adapter content graphs provide information on the percentage of adapters per read base. Adapters are generally found at the 3’‐end of the reads, because the sRNA sequence of interest is often shorter than the chosen sequencing length (e.g., 75 nt) (Figures 5a and 6a). High 3’ adapter content at the 5’‐end of the reads is indicative of the presence of adapter dimers, since the first sequenced base is the first nucleotide of the 3’ adapter and no sRNA was then sequenced.

In the milk EV experiment, a high percentage of the reads from samples 34–40 were characterized by high percentage of adapter content at the 5’‐end of the reads, which indicates full 3’ adapter sequence with no biological insert (Figure 5b middle panel and Figure S3). This matched with the observation of the peak identified at 23 min of migration in the electropherograms (Figure 5b left panel, samples 36 and 40). Importantly, the adapter content percentage in samples 34–40 rose proportionally with increasing percentage of read loss (Figure 5b and Figure S3). Adapter dimers content ranged from 0.3% to 78.4% (percentage of the total raw reads) (Table S4), with sample 40 showing the highest peak at 23 min in the electropherograms, the highest adapter dimer content at the 5’‐end (78.4%) of reads, as well as the most significant read loss (86.6%). In the EV‐enriched plasma GC experiment, the percentage of adapter dimers was much higher compared to the milk‐EV experiment. The percentage of adapter dimers in GC samples 1–3 varied between 28.9% and 35.4%, while this percentage further increased in GC samples 4–12 (69.4%–98.7%) (Table S5). This corresponds to the 88 bp peak in the electropherograms (Figure 6b and Figure S4). In EV‐enriched mice plasma samples, the percentage of adapter dimers varied between 36.9% and 71.7% (Table S7). In this case, the level of dimer sequences detected in the adapter content graph was small, due to the artificial fragmentation and mismatches/indels of the reads (Figure 6c and Figure S5). However, the presence of the peak at 88 bp and cutadapt result indicated adapter dimer contamination (Figure 6c and Figure S5). FFPE tissue samples were more heterogeneous regarding adapter dimers content, as it ranged from 15.8% to 91.6% (Table S8). The adapter dimers pattern in the FastQC graph was also observed in these samples (Figure 6d and Figure S6).

Hence, the FastQC adapter content graphs allowed to confirm the presence of adapter dimers and to evaluate the degree of contamination across samples from the same dataset.

#### 3.5.2 Overrepresented sequences

A standard library contains a diverse set of cDNA sequences. The presence of a single sequence being overrepresented in the library either means that it is a highly significant biological sequence, or that the library is contaminated with a redundant sequence. The ‛overrepresented sequences’ module in the FastQC report indicates the sequences which are present in the library with a percentage higher than 0.1% of the total sequences.

When overrepresented sequences were analysed across all datasets, we observed that some were full 3’ adapter sequences deriving from the sequencing of adapter dimers (sequences in blue, purple and yellow in Figure 5b; sequences in blue in Figure 6b–d ). The percentage of overrepresented sequences matched the percentage of adapter dimers content (Tables S5–S8 and Figures 5b and 6b–d middle panel) in the three experiments. Consequently, the high percentage of overrepresented adapter dimers in some samples resulted in the bioinformatics removal of a high number of uninformative reads, which caused a substantial read loss after pre‐processing.

#### 3.5.3 Per base sequence quality

During the pre‐processing of raw reads, adapter sequences are trimmed as well as regions of the reads with a quality score under a certain threshold (*q* < 20 in this study). Therefore, reads with a low quality score contribute to increase the percentage of read loss. The module ‛Per base sequence quality’ in the FastQC report shows the quality value of each sequenced base per position in the read.

**FIGURE 7:**
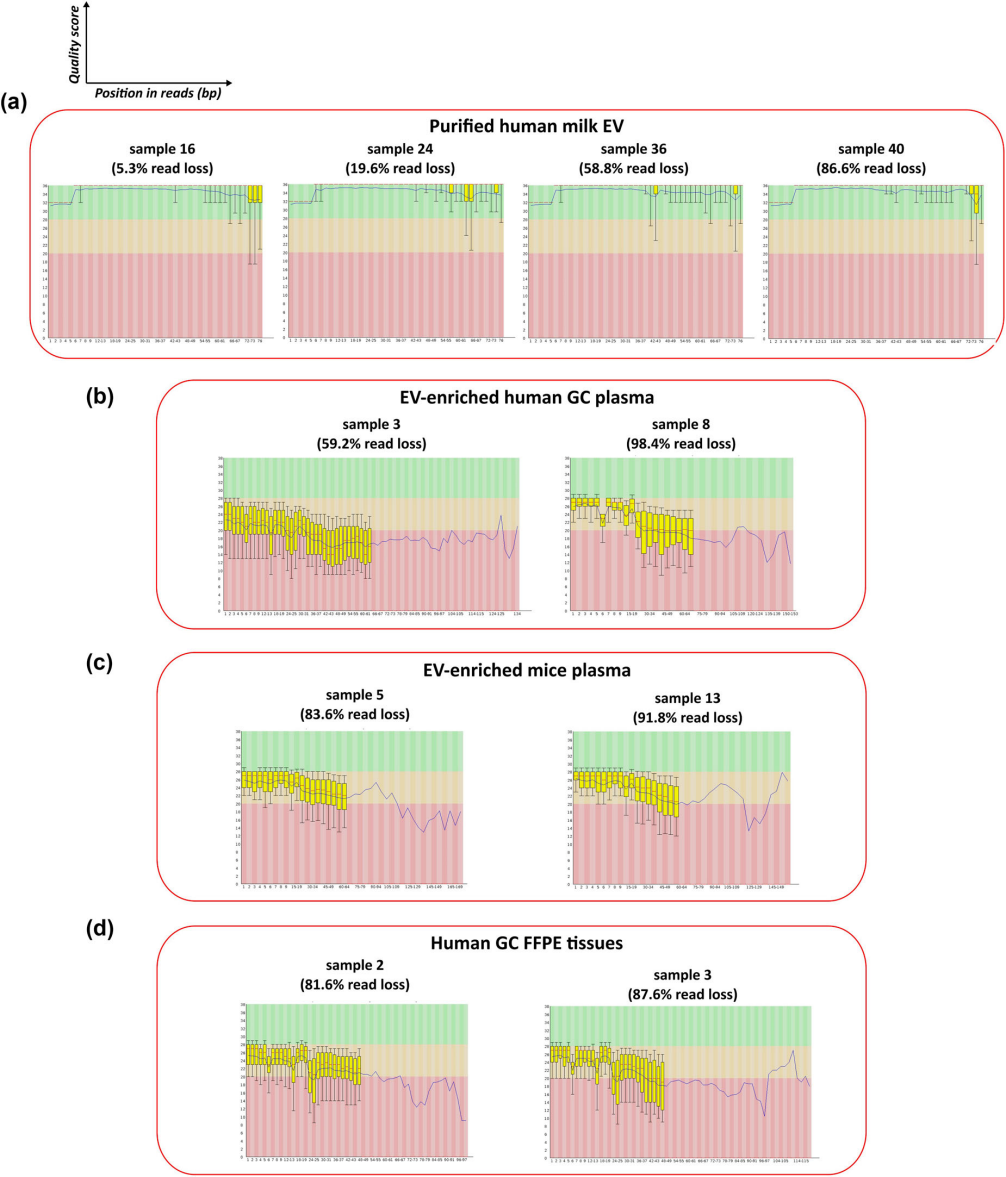
Quality of sequenced reads. Per base sequence quality before pre‐processing of reads in milk (a), GC (b), mice (c), and FFPE (d) experiments. Read bases or fragments with Q < 20 should be trimmed/removed in the pre‐processing of reads.

Generally, the read quality of the *Illumina* milk EV samples (Figure 7a) was higher than the quality of the *Ion Torrent* EV‐enriched GC plasma, mice samples and FFPE tissue GC samples (Figure 7b–d, respectively), therefore more read loss after pre‐processing was expected in the GC and mice experiments. However, within samples belonging to the same dataset, the quality of reads did not substantially differ between a sample with relatively low or high read loss, suggesting that the quality of reads did not determine the loss of reads within a certain group of samples.

In conclusion, the FastQC report is an effective tool to detect the presence of adapter dimer contamination in sequenced samples.

### 3.6 Analysis of short reads

The next parameter that may contribute to the read loss after pre‐processing is the removal of short reads. sRNA fragmentation or bad quality sequencing can produce short reads (<15 nt) that are not useful due to the high probability of alignment with many regions in the genome.

The amount of reads shorter than 15nt was calculated by cutadapt and converted into percentage of raw reads (Tables S5–S8). In the milk experiment, the percentage of short reads varied between 0.6% and 34.7%. Similar percentages were observed in the GC experiment, where this percentage varied between 0.4% and 20.8%. On the other hand, the mice and FFPE tissue GC experiments were characterized by samples with a general higher percentage of short reads, ranging from 14.0% to 46.6% and from 5.2% to 43.7%, respectively.

### 3.7 Read loss explained by high abundance of short reads and adapter dimers

**FIGURE 8:**
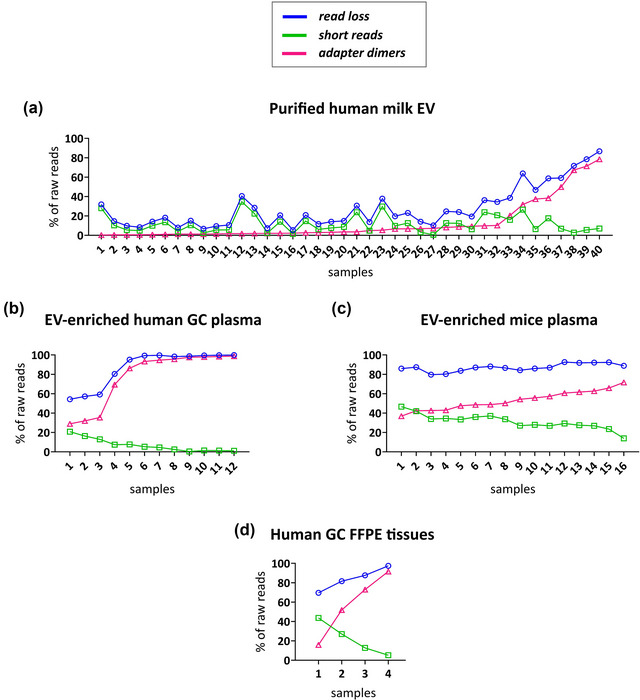
Contribution of short reads and adapter dimers to the percentage of read loss. Percentage of adapter dimers and short reads per sample are depicted (pink and green lines, respectively) for milk (a), GC (b), mice (c), and FFPE (d) experiments. Percentage of read loss is shown in blue.

Based on our analysis we assumed that the two major sources of read loss were caused by adapter dimer contamination and the abundance of short reads. Next, we evaluated their distribution across samples from the three experiments (Figure 8). In the milk‐EV experiment, we observed that the short read profile fitted the read loss profile in samples 1–32 (Figure 8a). However, in samples 33–40 adapter dimers were the main contributors for the high percentage of read loss (Figure 8a). In the EV‐enriched plasma GC samples, adapter contamination was the dominant cause of read loss after pre‐processing (Figure 8b), leading to the loss of almost 100% of the reads in some samples. Consistently, adapter dimers were also a major contamination in the EV‐enriched mice plasma samples (Figure 8c); however, this dataset was also characterized by a high percentage of short reads which, in combination with adapter dimers, resulted in the general severe read loss of about 80%–90% in all samples. In the case of FFPE tissue experiment, adapter dimers were identified as the major cause of read loss in 3 out of 4 samples of the dataset.

We found no correlation between the amount of RNA in ng and read loss, nor between the amount of RNA in ng and read loss. We observe some negative correlations, as between ng of sRNA and read loss in the milk experiment (Figure S7), which was the dataset with lower overall read loss. However, all the negative correlations which could associate higher read loss with lower RNA input were weak (<0.35). On the other hand, despite the apparent positive correlations between the amount of RNA in ng and adapter dimers, and between the amount of RNA in ng and read loss in the FFPE experiment, the low number of samples and the extreme degree of read loss, together with the illogical conclusion that more read loss is caused by more RNA quantity, turn these correlations very unmeaningful (Figure S7).

Overall, these data suggest that adapter dimer contamination is a typical issue in sequencing experiments with sRNA extracted from either purified EVs or samples enriched for EVs, and also FFPE tissues, independently from the sequencing platform used and the biological material. If not cleared, heavy contamination of sRNA libraries with adapter dimers ultimately lead to the failure of the sequencing experiment. Such sequencing files would therefore contain a tiny amount of reads of interest that may or may not reflect EV cargo. More importantly, even a low level of adapter dimer contamination can bias any downstream comparative analysis.

### 3.8 Batch effects generated by adapter dimer contamination: Technical variability surpasses biological variability

After identification and removal of non‐informative reads (adapter dimers) across all datasets, we proceeded with the bioinformatics pipeline for clean read alignment to the genome and gene expression quantification.

**FIGURE 9:**
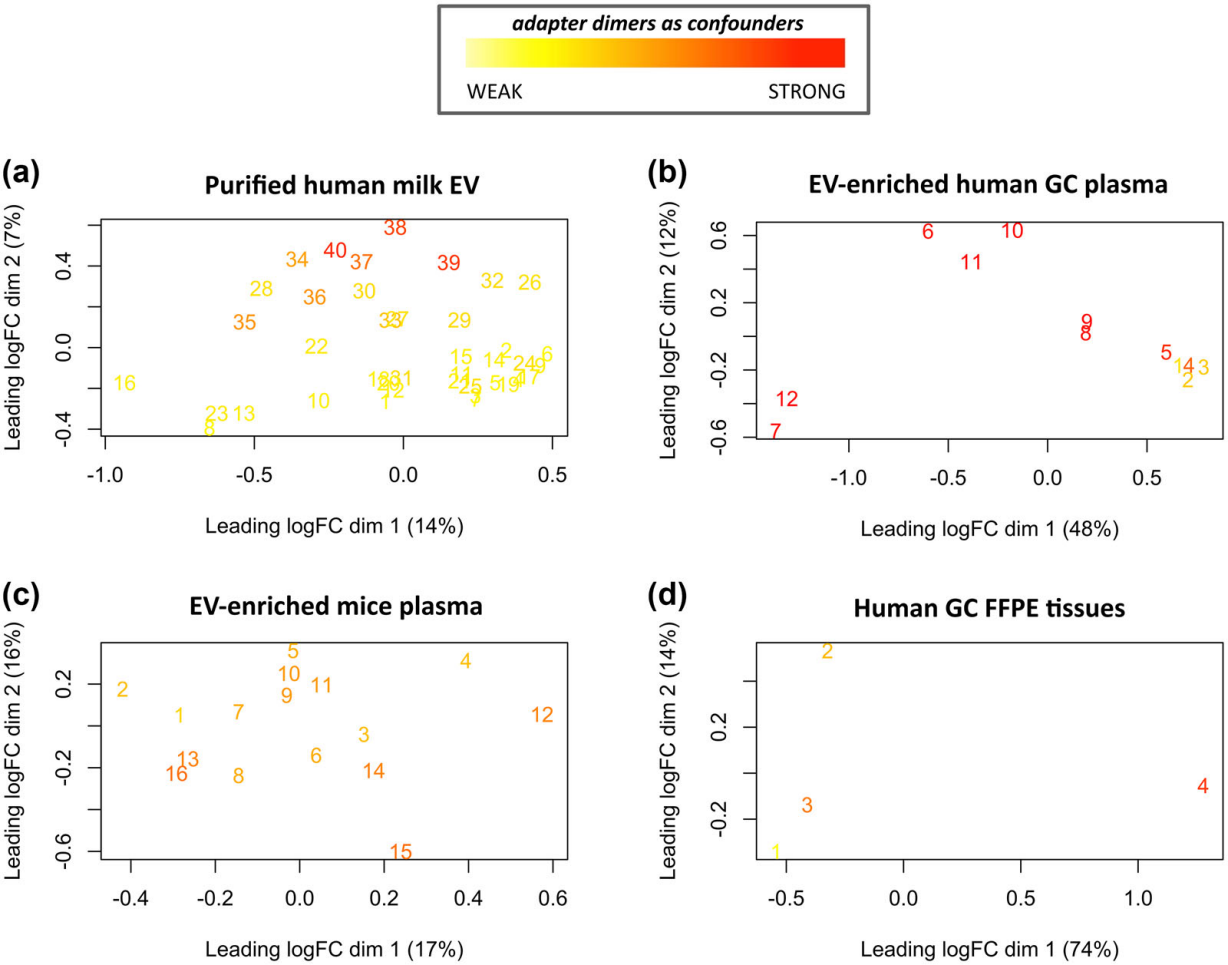
MDS plots showing sample dissimilarities of (a) milk EV, (b) EV‐enriched GC plasma, (c) EV‐enriched mice plasma and (d) FFPE tissue GC samples. The colour grade represents the order of the samples in a dataset based on adapter dimer contamination (from lowest to highest percentage of adapter dimers).

We used a multidimensional scaling (MDS) plot to visualise the level of similarity of individual samples across each dataset. Ideally, by using this approach different clusters based on biological differences could be distinguished. In the milk EV experiment, the MDS plot of the 40 samples showed two clusters (Figure 9a), however these clusters were not determined by biological differences but by the percentage of adapter dimer content. In fact, this technical variable (adapter dimer contamination) had a stronger impact on the clustering of samples than the biological variable (allergic vs. non‐allergic milk donors), thus generating a batch effect. Specifically, samples with relative high adapter dimer contamination (samples 34–40) were in the top part of the MDS plot (Figure 9a). Similarly, in the EV‐enriched plasma GC samples, a batch effect was observed, as samples with relatively low adapter dimer content (samples 1–3) clustered together (Figure 9b). As expected, no batch effect based on adapter dimer contamination could be identified in the MDS plot for EV‐enriched mice plasma samples (Figure 9c), since these samples had a more equally high adapter dimer contamination. Finally, no specific clustering was observed for FFPE tissue samples, probably because the number of samples was very low. Altogether, this analysis demonstrates that adapter dimer contamination may generate batch effects.

### 3.9 Preventing adapter dimer contamination to improve sRNA sequencing outcomes

The herein provided evidence that adapter dimer contamination can result in misleading effects in EV‐sRNA sequencing analyses and bias in data interpretation indicates the need for standardisation of methods and procedures to circumvent this contamination. We reviewed independent peer reviewed EV‐sRNA studies (Bouchareychas et al., 2021; Gad et al., 2020; Jia et al., 2021; Kupsco et al., 2021; Lucero et al., 2020; Nair et al., 2020; Robinson et al., 2021; Smith et al., 2020; Than et al., 2018; White et al., 2020) and selected those that applied pre‐sequencing adjustments leading to a reduction or avoidance of adapter dimers in sequencing outputs. Diverse EV sources were included in this collection of experiments and details on different methods for EV enrichment, RNA isolation, library preparation and sRNA sequencing are indicated in Table 1.

**TABLE 1:** Literature search of pre‐sequencing modifications that were shown to perform well in preventing adapter dimer contamination

Article	Citation	PMID	*n*	Source	EV isolation	RNA isolation	Library preparation	Pre‐sequencing modifications	Sequencing platform	Adapter dimers (%)
Glioma‐Derived miRNA‐ containing extracellular vesicles induce angiogenesis by reprogramming brain endothelial cells	(Lucero et al., 2020)	32075753	9	EVs from cultured human glioblastoma cells (neurospheres)	Differential ultracentrifugation	miRCURY RNA isolation Kit (QIAGEN)	NEBNext Small RNA (New England Biolabs)	Reaction volumes reduced to 1/5th Primer and adapter dilutions 1:6 Library purification (Zymo DCC‐5) Size selection 117‐135 bp (Pippin prep).	Illumina HiSeq 4000	Residual (0.2%)
Plasma extracellular vesicle miRNAs as potential biomarkers of superstimulatory response in cattle	(Gad et al., 2020)	33154526	12	EVs from plasma of heifers	exoRNeasy Serum/Plasma kit (QIAGEN)	exoRNeasy Serum/Plasma Kit (QIAGEN)	QIAseq miRNA (QIAGEN)	22 cDNA PCR cycles	Illumina NextSeq 500	Low (0%–8%)
Caveolin‐1‐driven membrane remodelling regulates hnRNPK‐mediated exosomal microRNA sorting in cancer	(Robinson et al., 2021)	33931969	6	EVs from cultured human advanced prostate cancer cells	Ultrafiltration and ultracentrifugation	TRIzol (Thermo Fisher Scientific)	NEBNext Small RNA (New England Biolabs)	Size selection (Perkin Elmer Labchip XT)	Illumina NextSeq	Low (0%–5%)
Human milk extracellular vesicle miRNA expression and associations with maternal characteristics in a population‐ based cohort from the Faroe Islands	(Kupsco et al., 2021)	33712635	372	Human breast milk‐EVs	ExoEasy Maxi kit (QIAGEN)	miRNeasy Serum/Plasma kit (QIAGEN)	HTG EdgeSeq miRNA WTA (HTG Molecular Diagnostics)	RNA cleanup (Zymo RCC‐5)	Illumina HiSeq 4000	No contamination
High glucose macrophage exosomes enhance atherosclerosis by driving cellular proliferation & hematopoiesis	(Bouchareychas et al., 2021)	34381972	8	EVs from cultured BMDM cells	Cushioned‐Density Gradient Ultracentrifugation	miRNeasy Mini Kit (QIAGEN)	NEXTFLEX Small RNA v3 (PerkinElmer)	DNAse + RNA cleanup (Turbo DNA‐free + Zymo RCC‐5) Size selection 140‐160bp (Gel purification)	Illumina HiSeq 4000	No contamination
Extracellular vesicles from Heligmosomoides bakeri and Trichuris muris contain distinct small RNAs	(White et al., 2020)	32659276	6	EVs from nematodes excretory/secretory products	Ultracentrifugation or SEC	miRNeasy Mini Kit (QIAGEN)	CleanTag Small RNA (TriLink Biotechnologies)	18 PCR cycles Adapter dilution 1:12	Illumina NovaSeq 6000	Residual (0%–0.2%)
Characterization of miRNAs in extracellular vesicles released from Atlantic salmon monocyte‐ like and macrophage‐like cells	(Smith et al., 2020)	33262769	8	EVs from salmon head kidney white blood cells	VN96 peptide (New England Peptide)	mirVana miRNA isolation kit (Invitrogen)	CleanTag Small RNA (TriLink Biotechnologies)	Double size selection (150–200bp) using Ampure XP beads	Ion Torrent Proton	No contamination
Sedentary and trained older men have distinct circulating exosomal microRNA profiles at baseline and in response to acute exercise	(Nair et al., 2020)	32587527	29	Exosomes from human plasma	ExoQuick (System Biosciences)	RNeasy Mini spin columns (QIAGEN)	NEBNext Small RNA (New England Biolabs)	15 PCR cycles Size selection (140‐160bp) using gel band manual excision.	Illumina MiSeq	Low (0%–2%)
Distinct extracellular RNA profiles in different plasma components	(Jia et al., 2021)	34234804	10	Exosomes from human plasma	ExoQuick (System Biosciences)	miRNeasy Micro Kit (QIAGEN)	CATS	15 PCR cycles Purification by AMPure XP beads	Illumina HiSeq 2500	Residual (0.1%)
Differential expression of keratinocyte‐derived extracellular vesicle miRNAs discriminate exosomes from apoptotic bodies and microvesicles	(Than et al., 2018)	30258405	18	EVs from cultured human keratinocytes	Differential centrifugation and microfiltration	TRIzol	Illumina TruSeq Small RNA	Size selection 145‐160bp (Gel purification)	ILLUMINA NextSeq 500	Residual (0%–1%)

There are several common features that seem to be advantageous for removing adapter dimer contamination. When the quantity of isolated RNA is predicted to be low, which is frequently the case with low input RNA sources such as EVs from blood samples or tissues (Crossland et al., 2023), the manuals for RNA isolation kits usually recommend diluting reaction and adapter volumes in order to concentrate the RNA of interest (Illumina, 2021). Along the same line, adjusting PCR cycles upwards is also often recommended and kit‐dependent. RNA clean‐up through purification steps (e.g., Zymo RCC kits ‐ Zymo Research) may refine sample RNA quantity and quality. Furthermore, library preparation kits can usually minimize or eliminate adapter dimers formation, namely: a) via the removal of the unbound 3’‐adapter excess before 5’‐adapter ligation (e.g., NEXTflex Small RNA‐Seq Kit v3 ‐ Bioo Scientific); b) through an early inclusion of the RT primer (e.g., NEBNext Small RNA library preparation kit ‐ New England Biolabs); c) through quantitative nuclease protection assay technology (e.g., HTG EdgeSeq miRNA WTA ‐ HTG Molecular Diagnostics); d) by Capture and Amplification by Tailing and Switching (CATS, Diagenode), e) by using chemically modified adapters (e.g., preadenylated 3’‐adapters in QIAseq miRNA Library Kit ‐ QIAGEN; and CleanTag Small RNA Library Preparation Kit ‐ Trilink Biotechnologies), or f) by using adapters with randomized ends (e.g., NEXTflex Small RNA‐Seq Kit v3 ‐ Bioo Scientific).

Once the library preparation process is finished and before sequencing, it is possible to enrich the libraries as much as possible for the desired product. This may be achieved by size selection for the length of the sRNA of interest only. For this, bead‐based methods included in several library preparation kits (e.g., AMPure XP kit ‐ Beckman Coulter), gel electrophoresis (e.g., with Pippin systems ‐ Sage science; and LabChip XT ‐ Caliper) or a combination of both, have been shown to be beneficial (Head et al., 2014).

The library preparation kits, as presented in Table 1, were developed based on different approaches, in order to avoid adapter dimer formation and other types of contamination, especially when sequencing low input material. In fact, when we inspected the adapter dimer content in the selected studies with six or more samples sequenced, we could not find more than 10% of adapter dimers in a single sample, and in some cases, adapter dimers were completely absent. This analysis demonstrates that it is possible to reduce or eliminate adapter dimers in sRNA sequencing experiments.

## 4 DISCUSSION

NGS analysis of sRNA has attracted much interest in the EV field, as it allows gaining insight on which sRNA are shuttled by EVs, their potential effects on recipient cells and their possible use as disease biomarkers. However, in order to obtain robust research outcomes that allow reproducibility and potential clinical applications, there is a need for standardisation and optimisation of procedures. Several benchmarking studies have been performed for EV isolation, EV‐RNA extraction, as well as EV purification to remove co‐isolated RNA‐containing structures (Mateescu et al., 2017; Hill et al., 2013; Witwer et al., 2013). In contrast, challenges related to the sRNA sequencing procedure, associated quality control steps and bioinformatics analysis are not often addressed. A clear example is adapter dimer contamination in sequencing libraries, albeit recognised more than a decade ago vastly disregarded, creating a large potential for bias in data analysis and interpretation.

The main aim of this paper is to explore the quality control data generated across all steps of the sRNA sequencing workflow and subsequent bioinformatics analysis, in order to identify flags for intervention, correction or to prevent proceeding downstream analysis. Therefore, we used four sRNA sequencing datasets obtained from different biological sources by different experimental procedures. Interestingly, a common feature in all these datasets was a sizeable or variable drop in the number of reads after pre‐processing of sRNA sequencing data. Through a step‐by‐step analysis of the quality controls for RNA isolation, library preparation and sequencing processes, we found that adapter dimer contamination was the major cause for marked read loss, which resulted in technical variations between different samples within datasets and in the generation of batch effects.

When we performed the quality control checks on isolated RNA of individual samples of a dataset, we did not find a correlation between high amount of adapter dimers and relatively low sRNA quantity/quality. The observed inter‐sample variation in the amount of isolated sRNA could be caused by technical aspects related to the methods and kits used for EV and RNA isolation (Gaarz et al., 2010), or by biological aspects related to heterogeneity in milk, plasma or tissue donors or in EV‐subsets (Martínez‐González et al., 2020; Willms et al., 2016). The sorting of RNA into different EV subsets is not yet fully understood (Fabbiano et al., 2020), which makes EVs an uncertain source of RNA, as opposed to cells (Olivares et al., 2020).

The observed lower quantity of isolated RNA from EV‐enriched plasma samples (GC and mice experiments) compared to purified milk EVs could be associated with the overall increase in adapter dimers in the sequencing of plasma material compared to milk. Because of the potential influence of RNA quantity on sequencing results, it is deemed critical to evaluate the quantity and quality of the isolated RNA by means of sensitive methods such as NanoDrop, the Qubit RNA assay or Bioanalyzer/Fragment Analyzer/Tapestation softwares (Fischer & Deindl, 2021).

Another important consideration when working with EV‐sRNA is the great difficulty to define RNA quality. Evaluation of RNA quality can be achieved by examining the RIN (RNA Integrity Number), calculated with the concentrations of 18S and 28S rRNAs. However, when working with EVs, RIN cannot be calculated because the concentrations of 18S and 28S rRNAs are not consistent among EVs (Crescitelli et al., 2013) and rRNAs are not always isolated (e.g., when only sRNA < 200 nt are isolated) (Cheng & Hill, 2017). Instead, the Bioanalyzer profiles can be used to identify potential degradation of RNA. Even though this provides only an approximate insight into the RNA quality, it is an important step when suboptimal collected, stored or (partially) degraded material is used as RNA source, for example clinical blood samples or paraffin embedded tissues. Furthermore, it is not known whether RNA degradation in low quality RNA samples occurs uniformly or not, potentially influencing selective transcripts quantification (Gallego Romero et al., 2014).

cDNA electropherograms provide the size distribution of the library of interest and potential sources of contamination. Of particular relevance, and a significant outcome of the current study, cDNA electropherograms allowed the detection of adapter dimer contamination in sequencing experiments. Importantly, inspection of cDNA libraries via electropherograms is the last quality control step taking place in the sequencing facility prior sequencing, thus allowing possible corrections/solutions for the removal of adapter dimers reviewed in Table 1. An additional method for the detection of adapter dimer contamination is the analysis of the ‛adapter content’ graph in the FastQC report, in which a percentage of adapter content above 0% from base 1 should be considered as a warning bell (Andrews, 2010). Furthermore, in the FastQC module ‛overrepresented sequences’ we could identify full 3’ adapter sequences at a very high percentage in the majority of EV‐enriched plasma samples. In contrast to the analysis of the cDNA electropherograms, the FastQC report modules allow for a post‐sequencing detection of adapter dimers. This provides a quality check for a last bioinformatics strategy to correct the potential contamination ahead of downstream analysis of the data. Notably, post‐sequencing bioinformatics approaches can ameliorate usable data, but will not solve the problem, as sequencing data contain a large number of reads corresponding to adapter dimers, jeopardising the biological meaning of the study. Consequently, high number of uninformative reads are lost due to the proper bioinformatics removal in the pre‐processing step. Careful inspection of cDNA electropherograms before proceeding with sequencing is thus strongly preferred and should be included in the RNA‐seq workflow.

Additionally, when adapter dimer contamination arises in a subset of samples in a dataset, it is likely that a batch effect is generated, threatening biological interpretation. MDS plots are used to detect biological or experimental differences between samples by sample clustering. The MDS plots generated with our datasets showed that samples can cluster based on abundance of adapter dimers, indicating that adapter dimer contamination was able to generate a batch effect, which may produce a bias in the downstream biological analysis. Such bias can be the cause of the lack of reproducibility across studies using similar experimental setups and biological materials.

Despite the problems that adapter dimer contamination may generate in EV‐sRNA sequencing data analyses, it is relatively easy to prevent them by not allowing adapter dimers to be sequenced, thus resulting in larger number of mapped and usable reads. In fact, becoming aware of adapter dimer contamination is enough to determine its extent and decide whether it is feasible and trustworthy to proceed with downstream analysis. In general, quality controls as shown in this study and indicated in RNA‐seq good practice papers (Aparicio‐Puerta et al., 2020; Su et al., 2014) are essential for robust and reproducible EV‐sRNA analysis. This is of utmost importance especially when the aim of the sequencing research is exploitation of EV cargo in clinical diagnostics and biomarker discovery. Furthermore, quality control steps should be reported in the Methods section, even when using available pipelines that automatically execute the full analysis. For instance, some studies still do not clearly specify the adapter sequences used, which critically undermines the reproducibility of the data (Zhong et al., 2019). Moreover, depositing data in public repositories to foster satisfactory data review and sharing should be encouraged.

In conclusion, in this study we dissected quality control steps in sRNA sequencing experiments and presented a workflow for robust EV‐sRNA analysis that allows monitoring quality parameters, corrections for deviations and proceeding to sequencing with a greater likelihood of success.

## AUTHOR CONTRIBUTIONS

**Joaquín J Maqueda**: computational analysis; data curation; writing – original draft; review & editing. **Alberta Giovanazzi**: data curation; figures design; writing – original draft; review & editing. **Ana Mafalda Rocha**: plasma sRNA sequencing work. **Sara Rocha**: GC experimental work. **Isabel Silva**: mice experimental work. **Nadine Saraiva & Nuno Bonito**: GC patients’ collection and stratification. **Joana Carvalho**: GC‐related experimental work. **Luis Maia**: conceptualisation and supervision of the mice project. **Marca H.M. Wauben**: funding acquisition; study conceptualisation; supervision of the milk project; writing – review & editing. **Carla Oliveira**: funding acquisition; study conceptualisation and design; supervision of GC‐related projects; writing – review & editing. All authors: review; editing and approval of the manuscript.

## CONFLICT OF INTEREST STATEMENT

The ACCESS study was partially funded by Nutricia Research (Uppsalalaan 12, 3584 CT, Utrecht, The Netherlands) as part of a partnership grant of the Dutch Technology foundation STW (Project STW 11676: Exosome‐based biomarker profiling of breast milk: Definition of predictive immunomodulating biomarker profiles for the management of allergic disease development in infants).

## Supporting information

Supporting Information

Supporting Information

Supporting Information

Supporting Information

Supporting Information

Supporting Information

Supporting Information

Supporting Information

Supporting Information

Supporting Information

Supporting Information

Supporting Information

Supporting Information

Supporting Information

Supporting Information

## Data Availability

The small RNA sequencing data generated in the ACCESS study is accessible via NCBI GEO at the accession number GSE216498. As sharing data from biological samples from gastric cancer patients may compromise legal requirements, these data will not be shared. All mouse data from the current study can be made available after review of requests for overlap with ongoing analyses and according to site‐specific policies for data access. Data sharing requests should be sent to LM: luismaia.neurologia@chporto.min‐saude.pt.
