# Supplementary figures and images for: Adapter dimer contamination in sRNA‐sequencing datasets predicts sequencing failure and batch effects and hampers extracellular vesicle‐sRNA analysis
Source: J Extracell Biol. 2023 Jun 11;2(6):e91. doi: 10.1002/jex2.91 (PMC11080836; doi:10.1002/jex2.91)

**a****EV-enriched human GC plasma**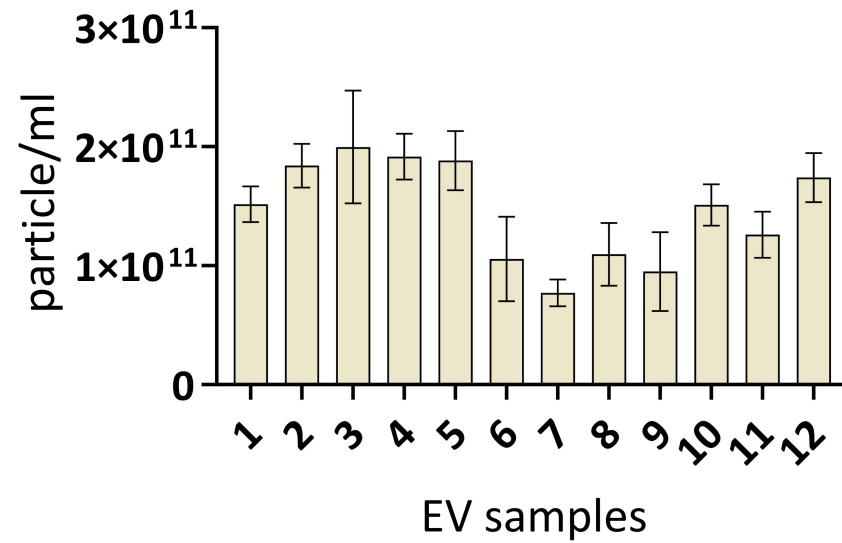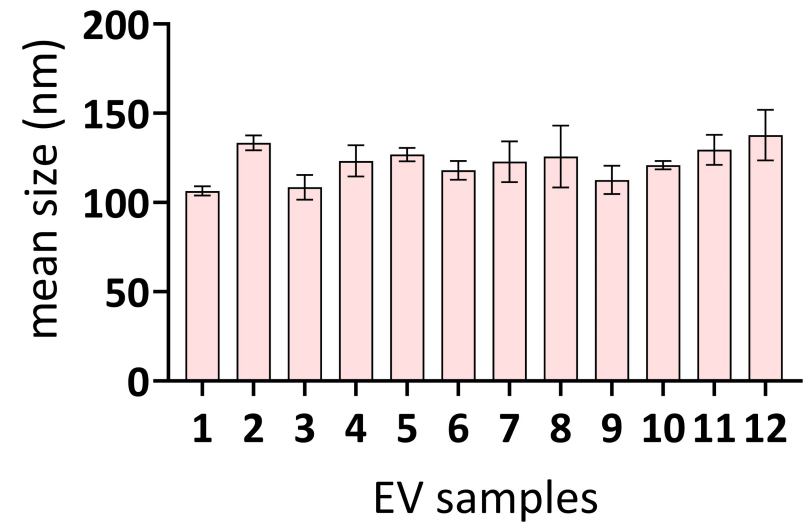**b****EV-enriched mice plasma**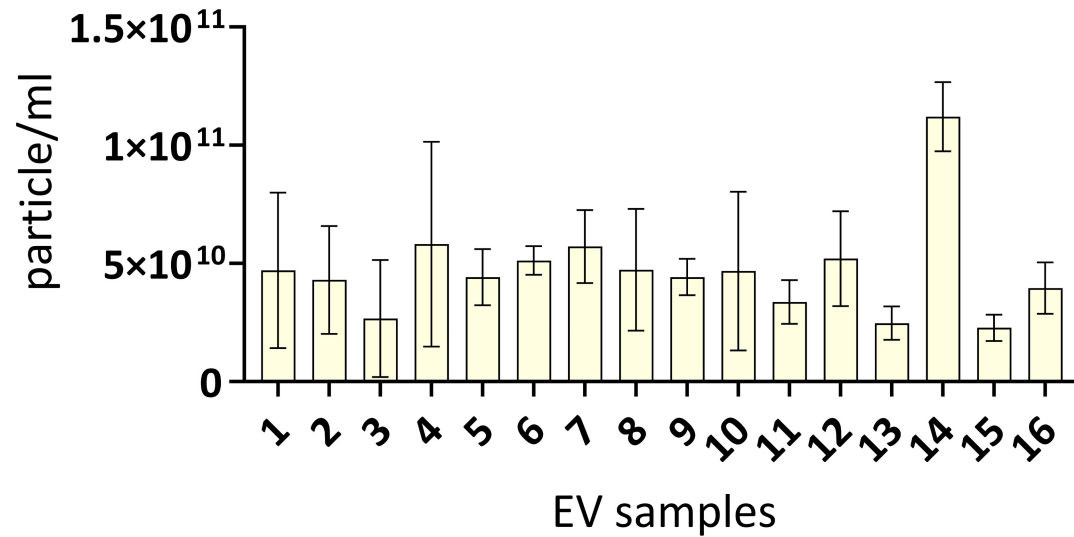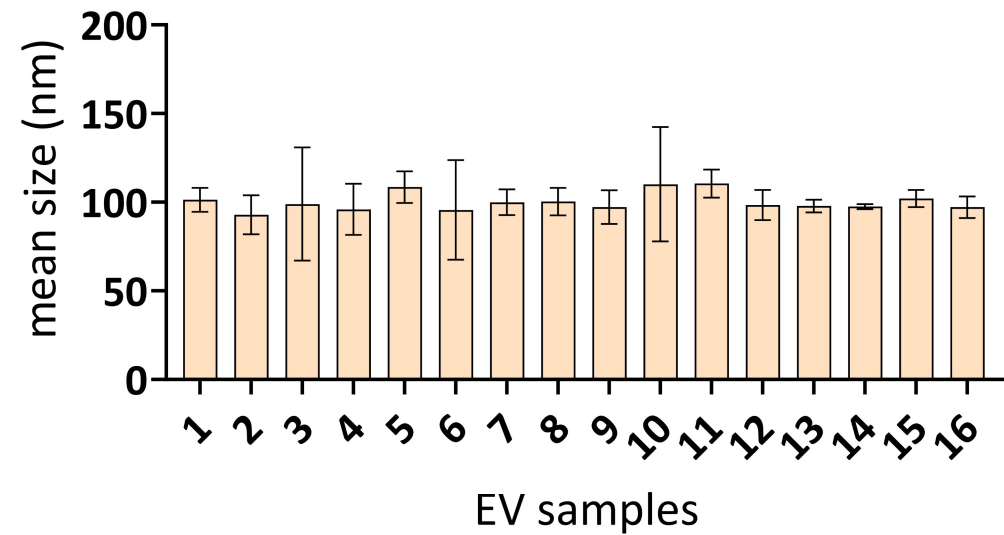

Supplement: Supplementary file 1 — Supporting Information [file JEX2-2-e91-s014.pdf]

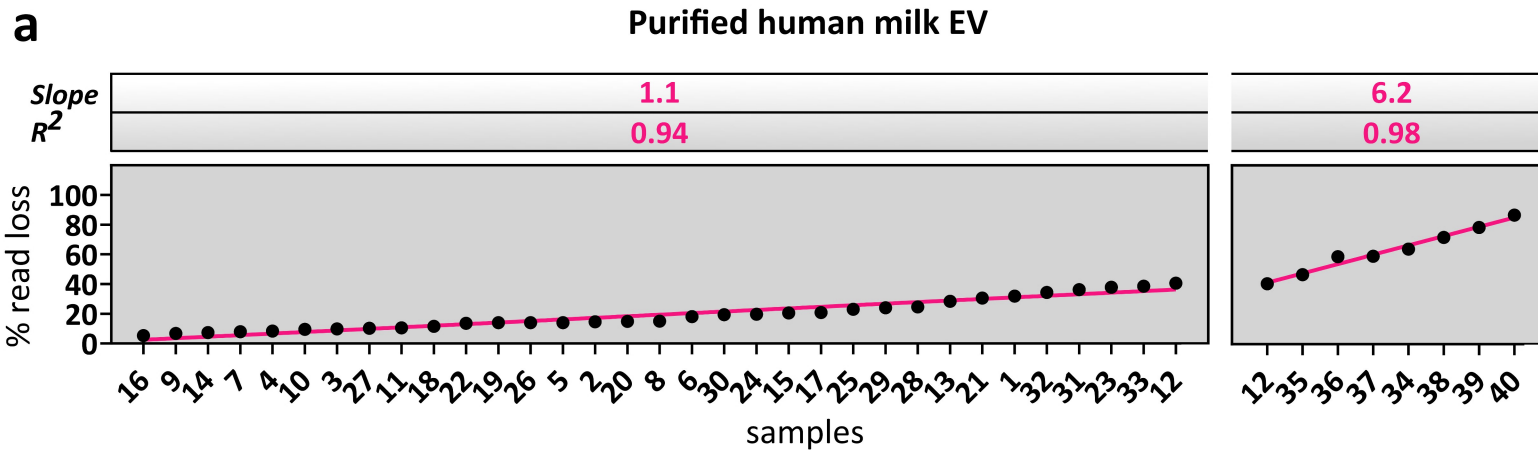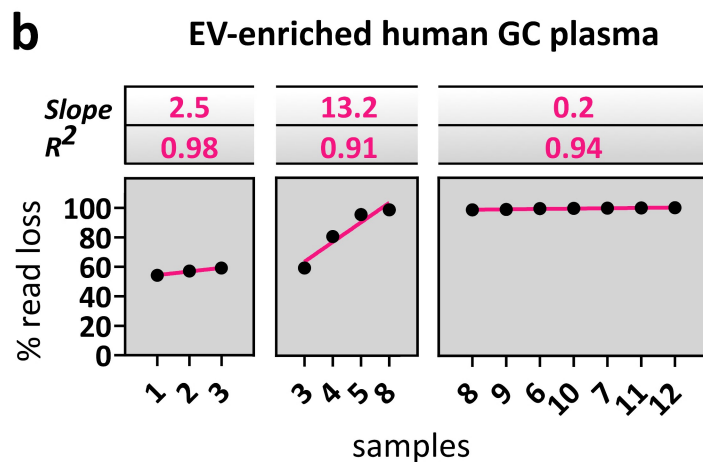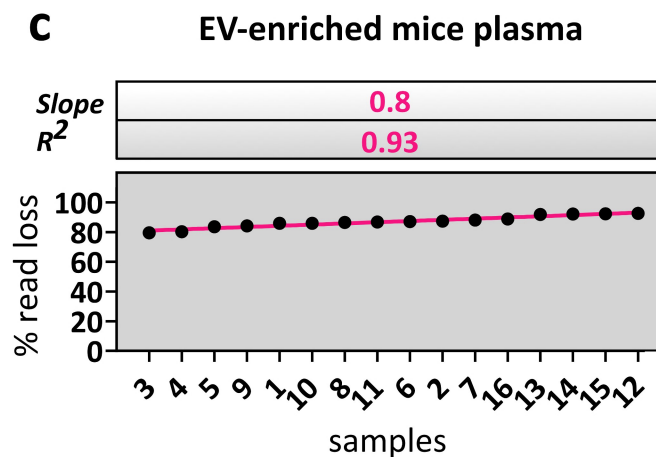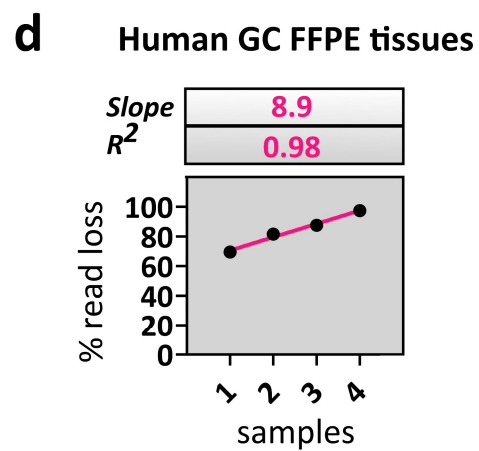

Supplement: Supplementary file 2 — Supporting Information [file JEX2-2-e91-s009.pdf]

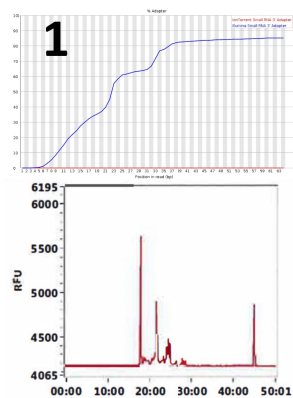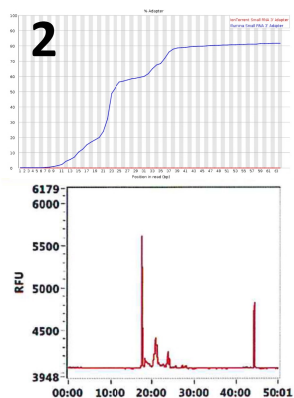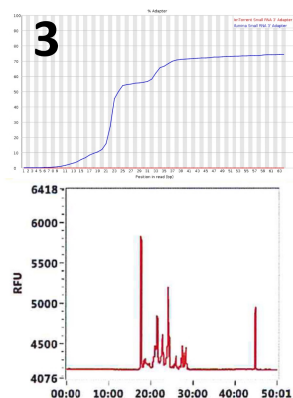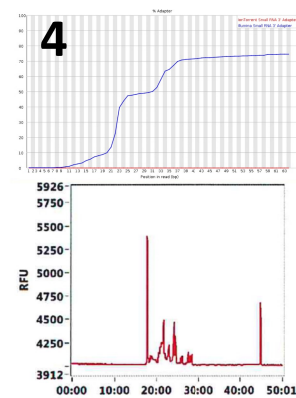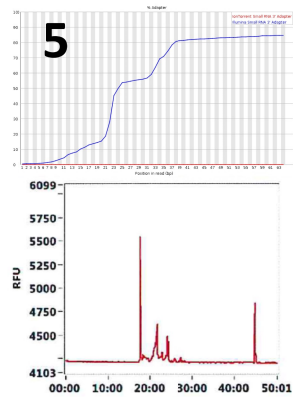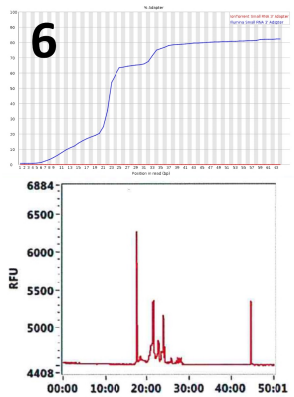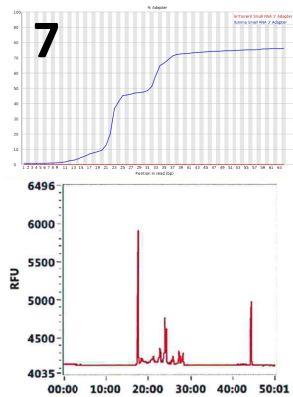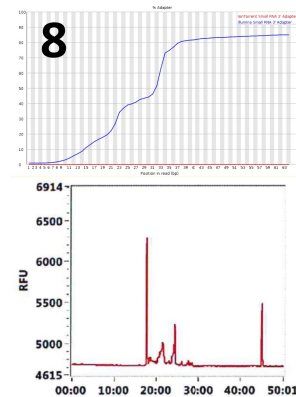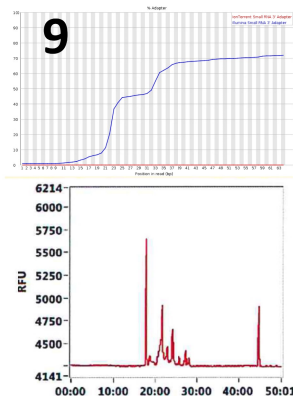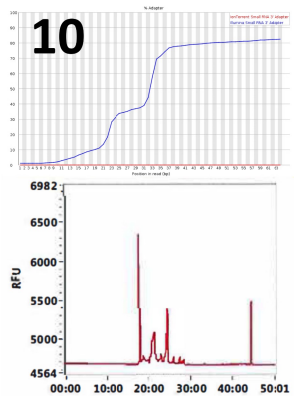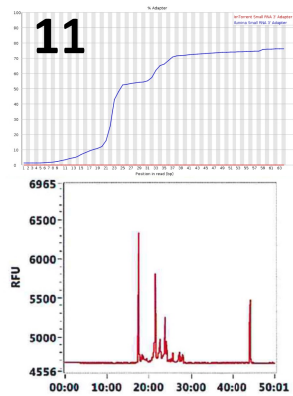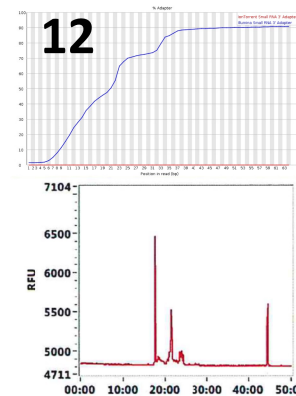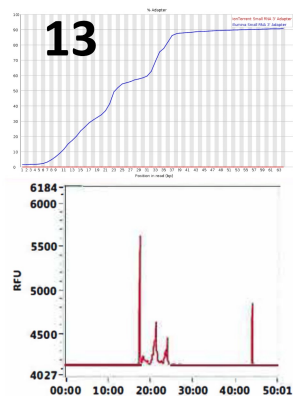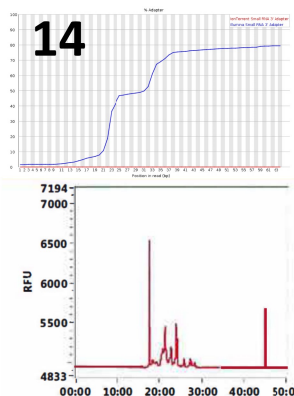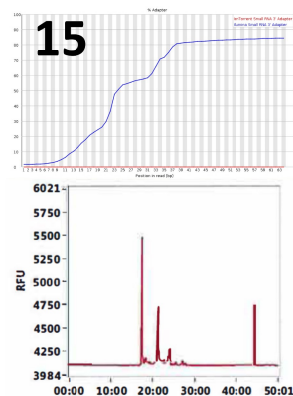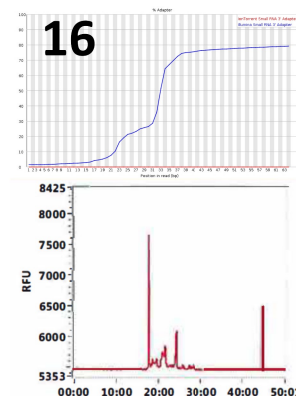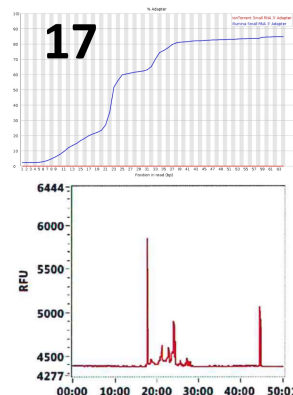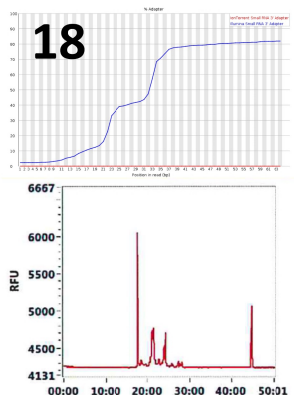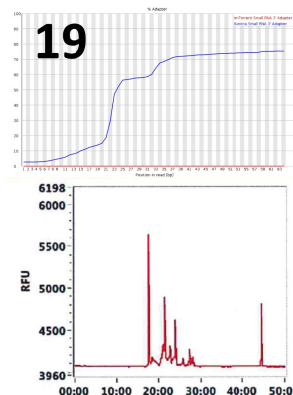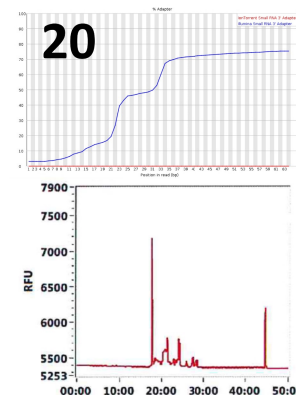

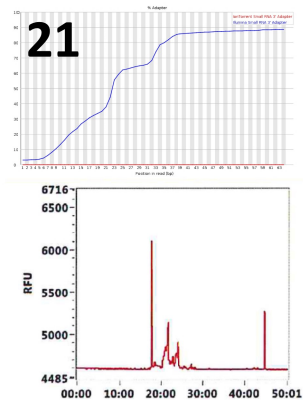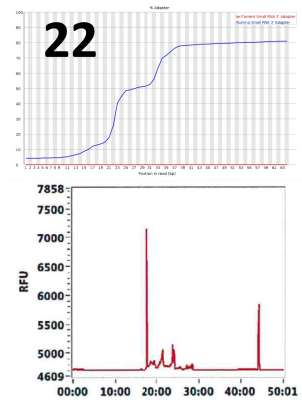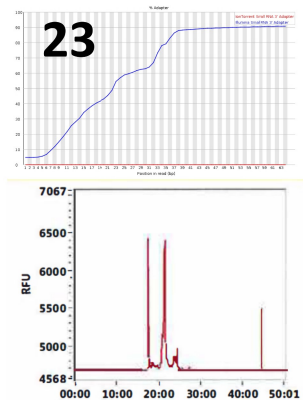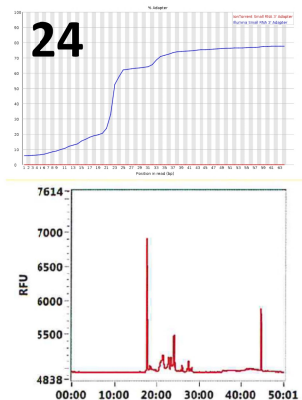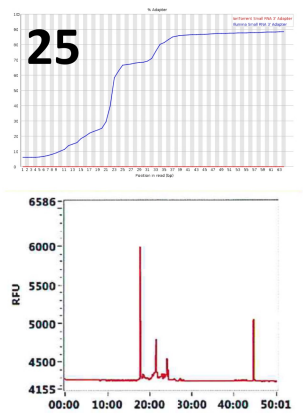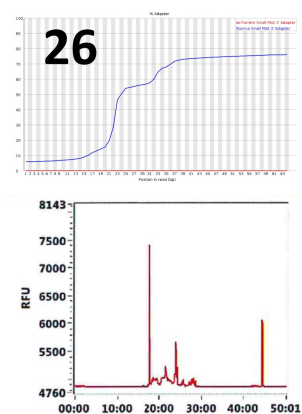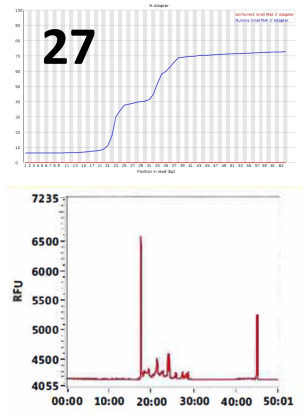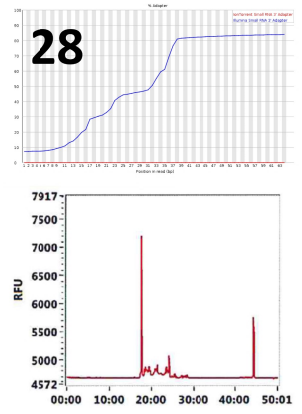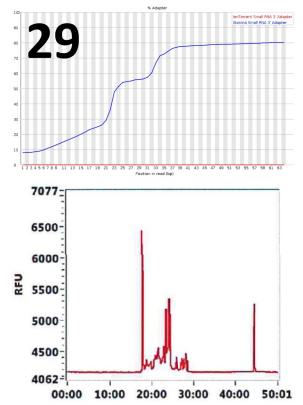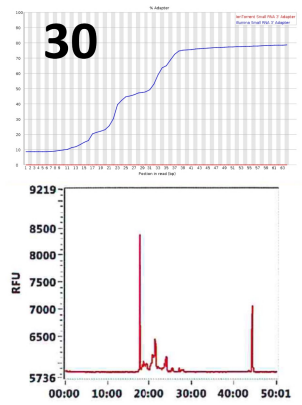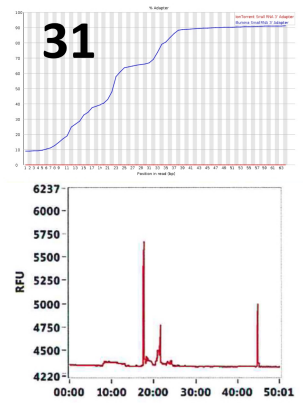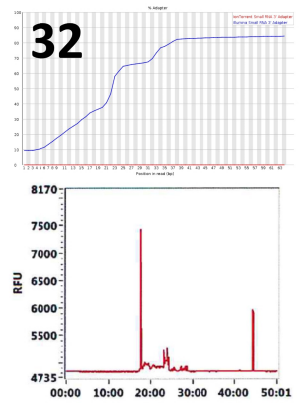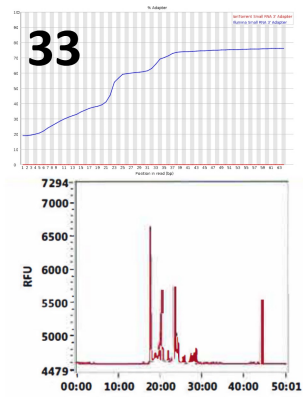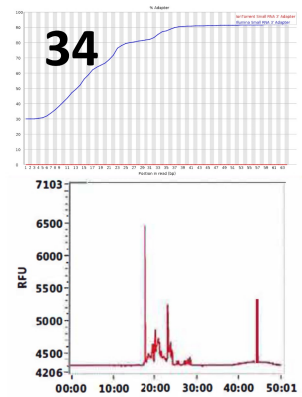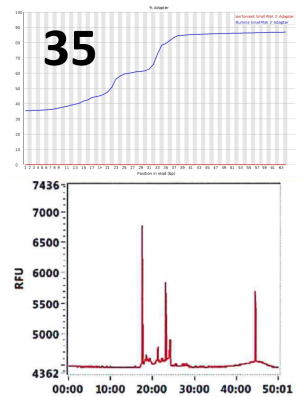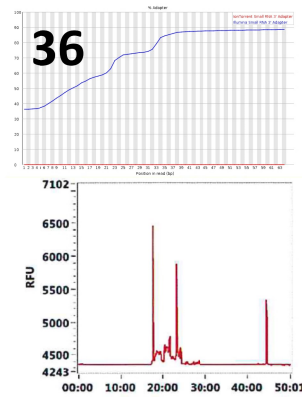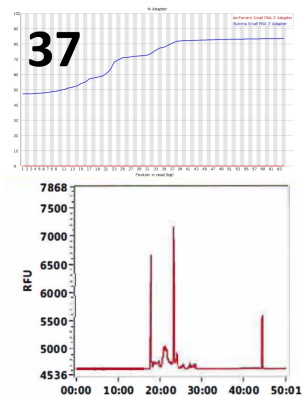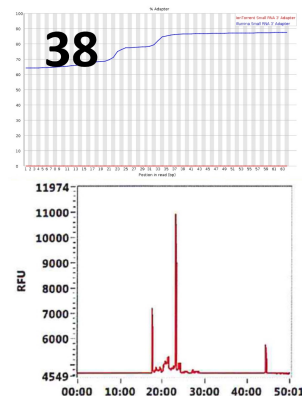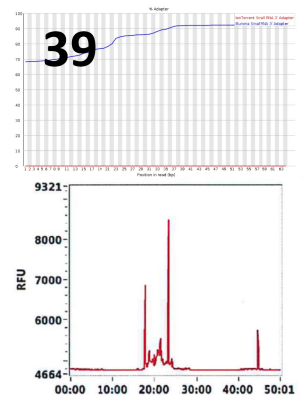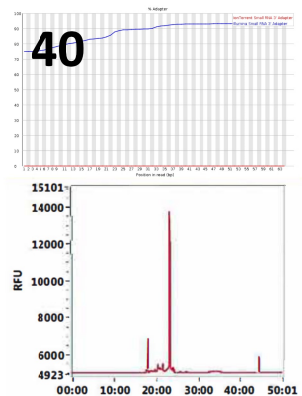

Supplement: Supplementary file 3 — Supporting Information [file JEX2-2-e91-s010.pdf]

1

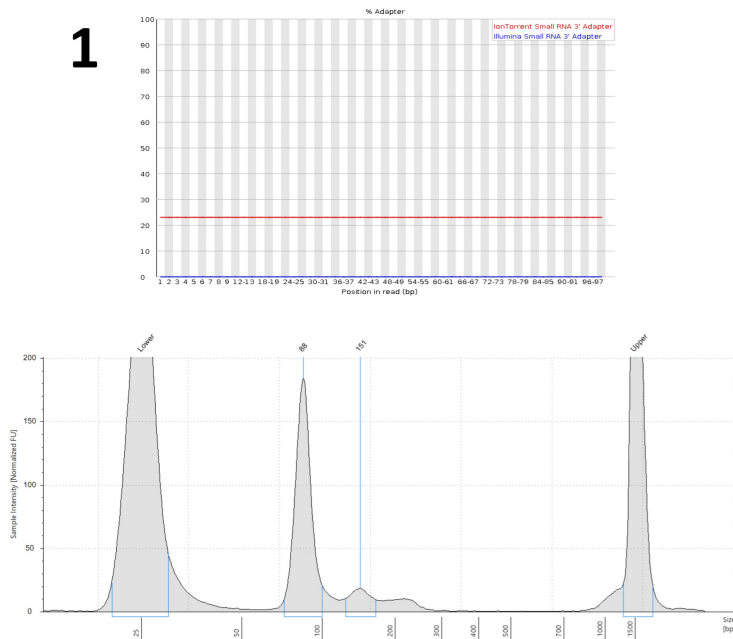

2

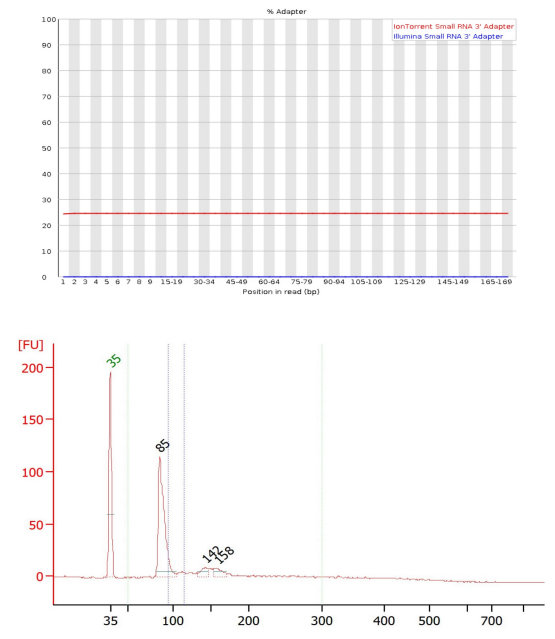

3

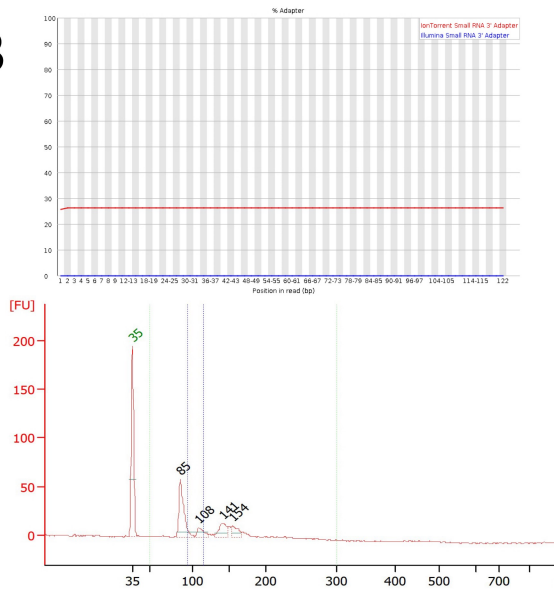

4

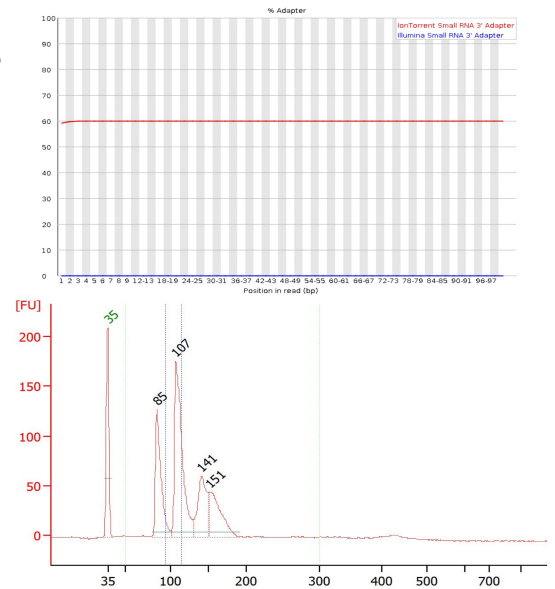

5

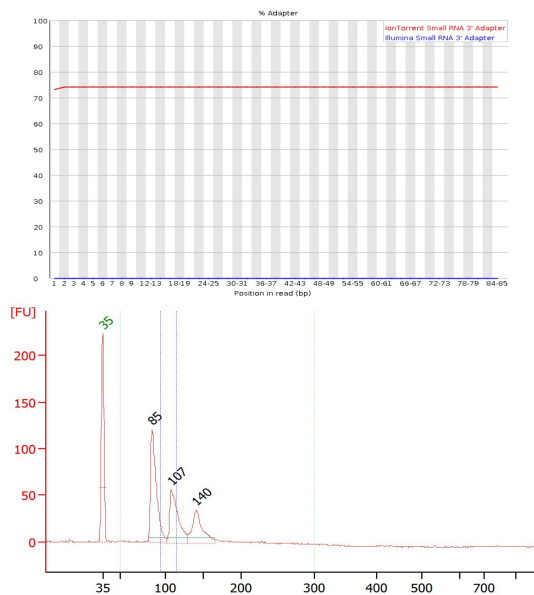

6

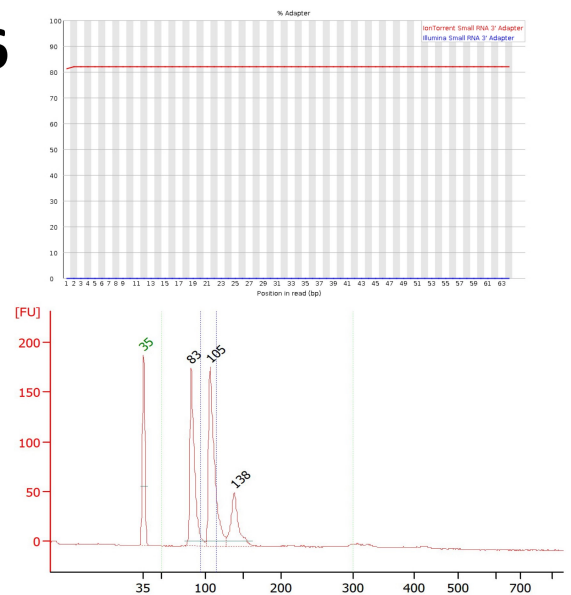

7

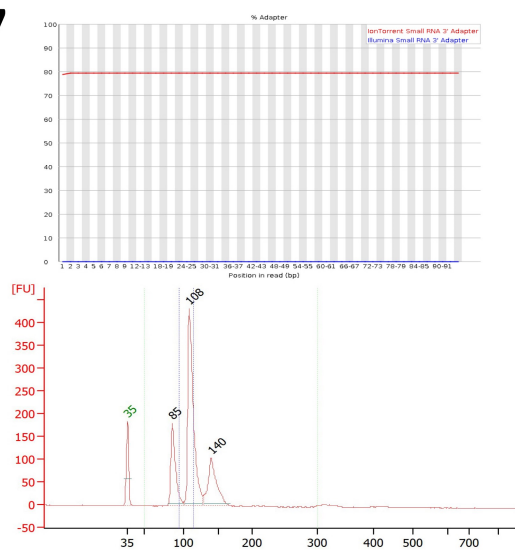

8

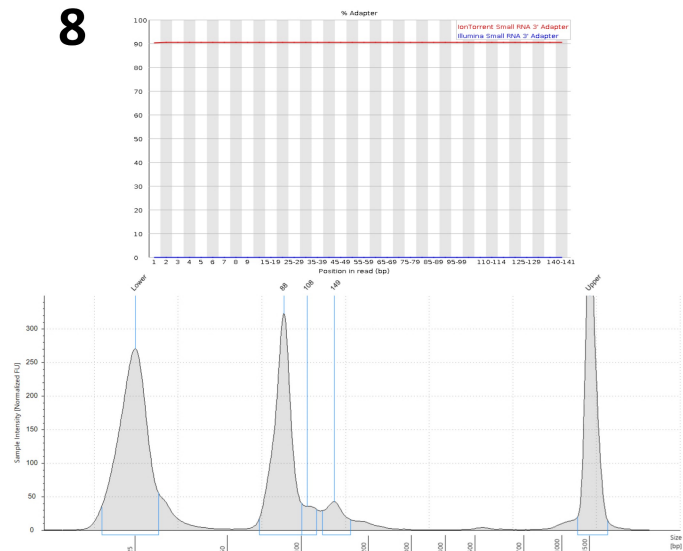

9

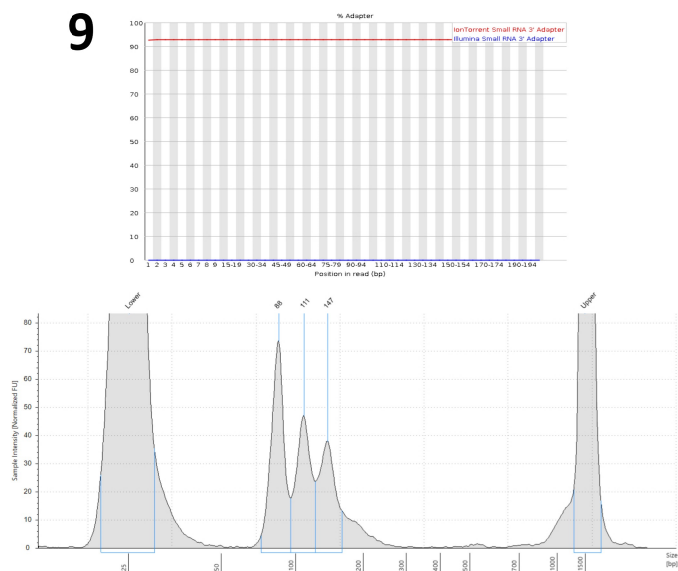

10

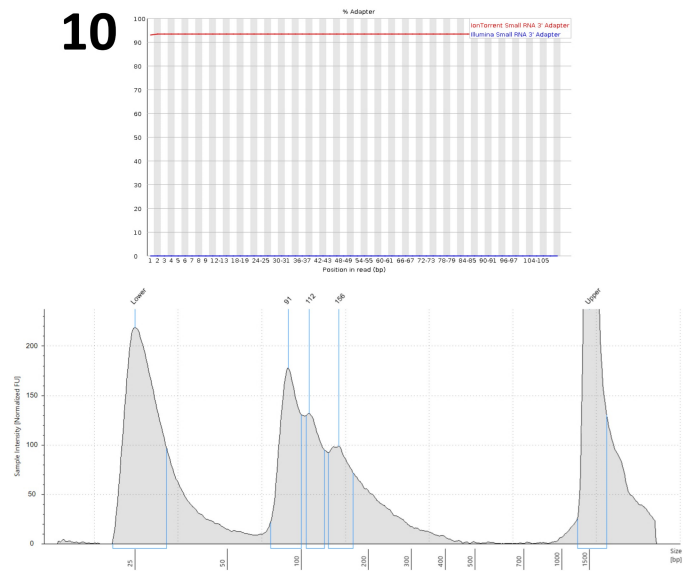

11

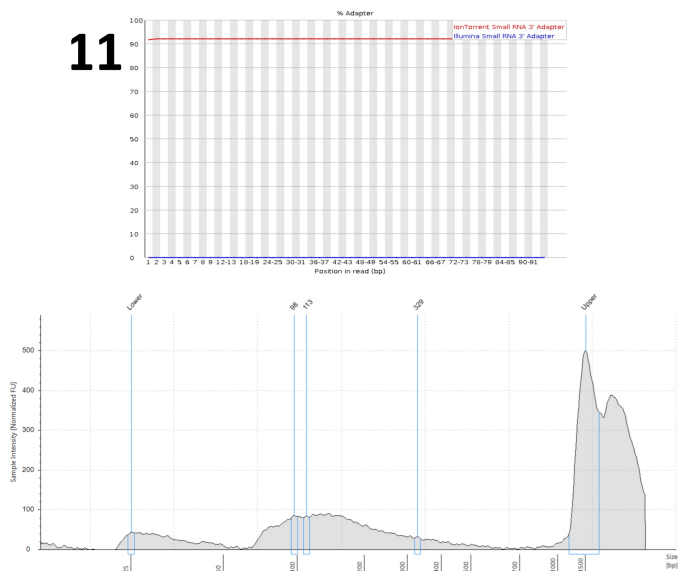

12

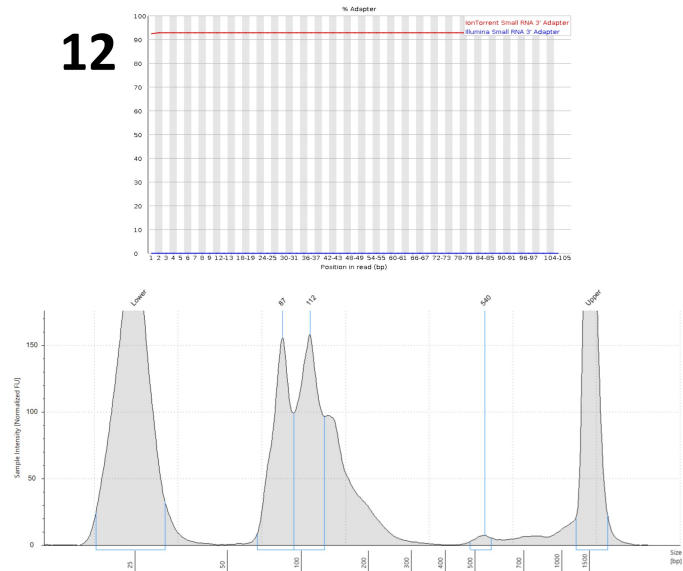

Supplement: Supplementary file 4 — Supporting Information [file JEX2-2-e91-s003.pdf]

1

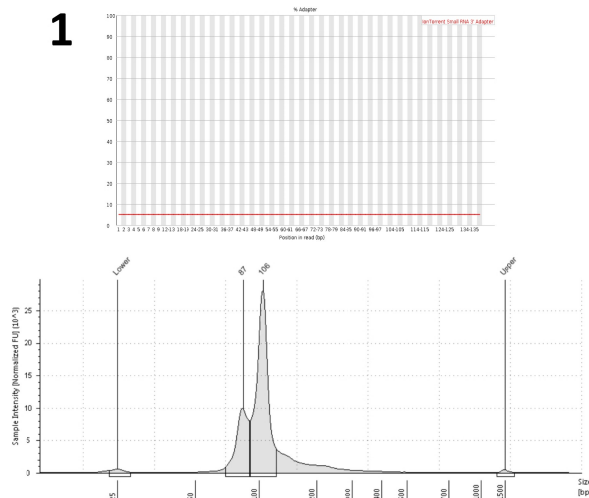

2

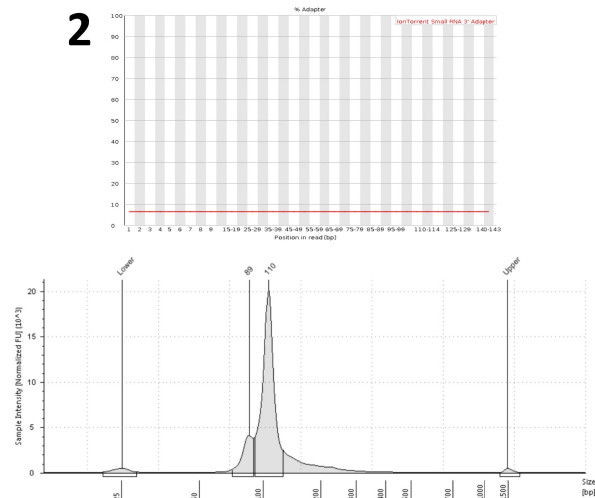

3

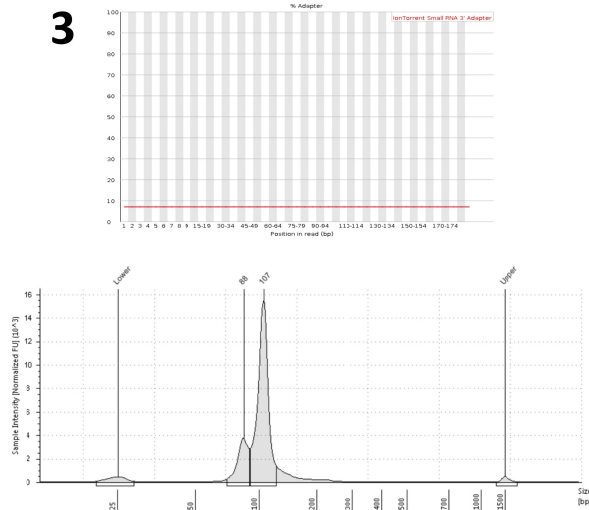

4

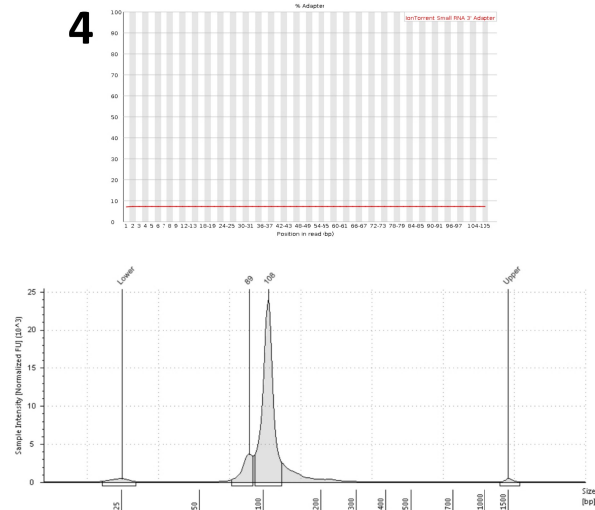

5

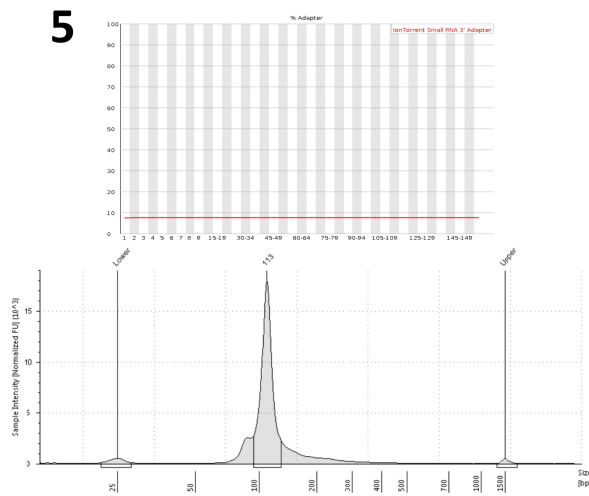

6

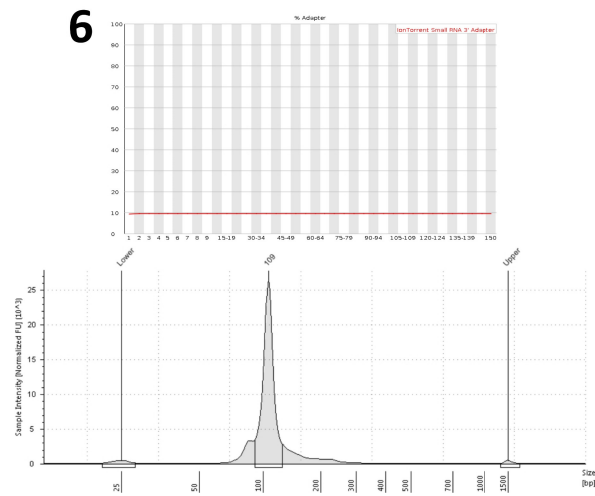

7

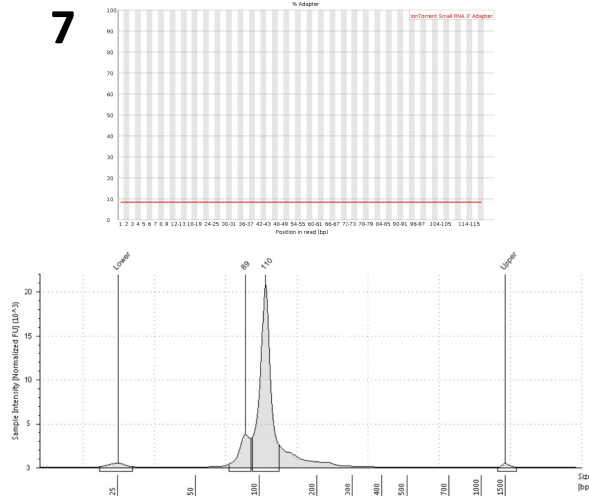

8

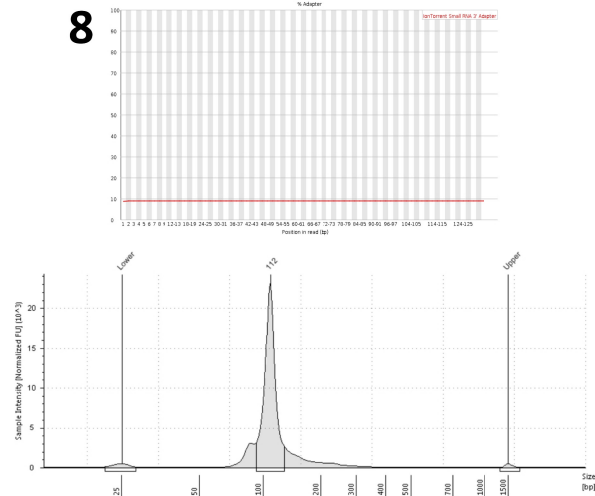

9

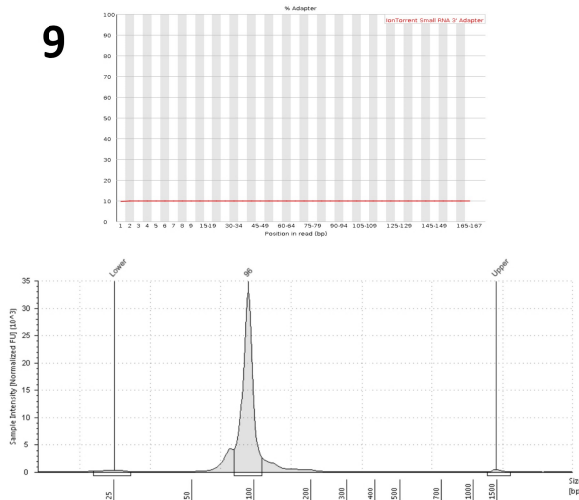

10

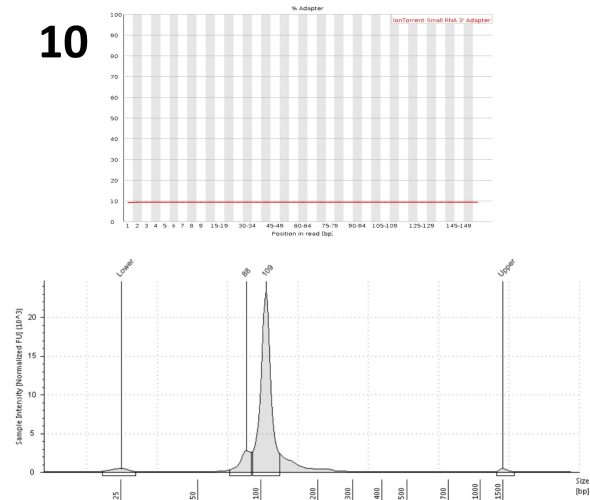

11

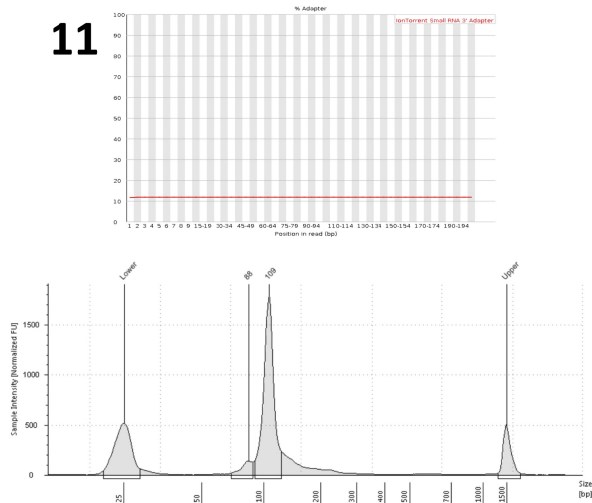

12

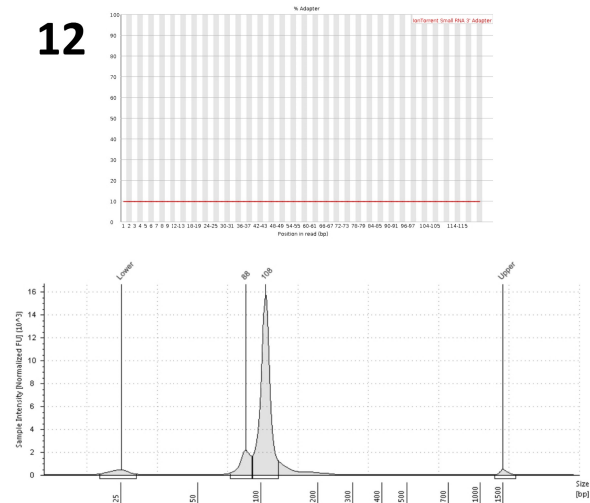

13

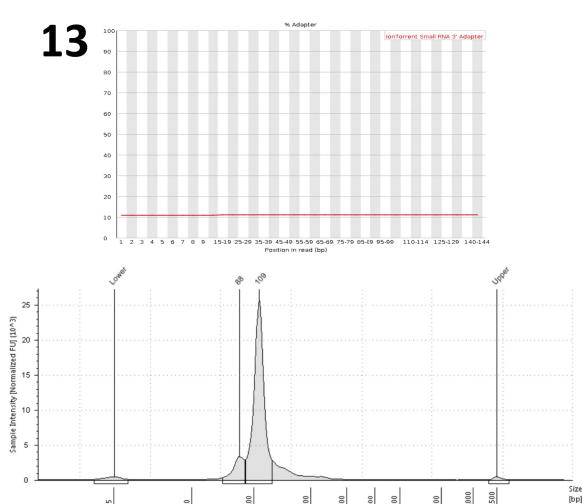

14

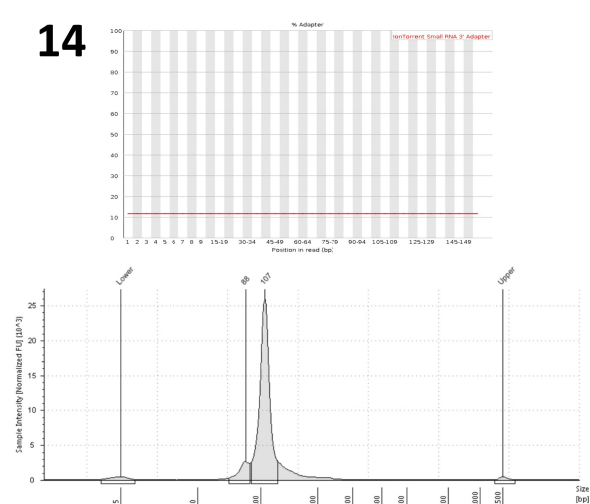

15

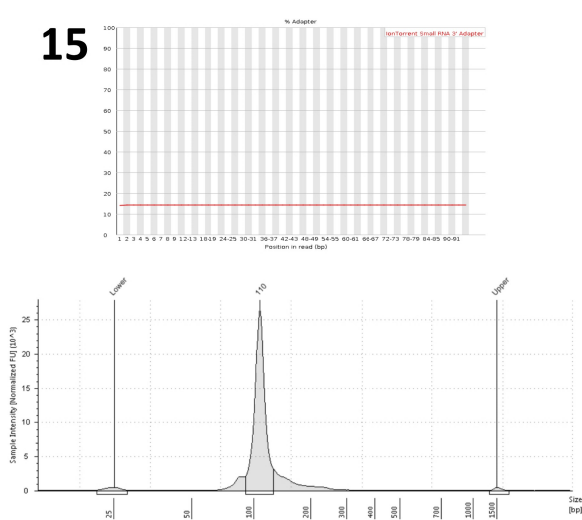

16

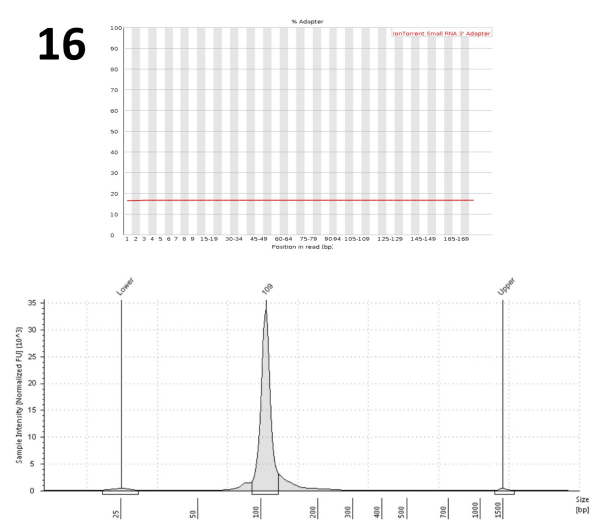

Supplement: Supplementary file 5 — Supporting Information [file JEX2-2-e91-s002.pdf]

1

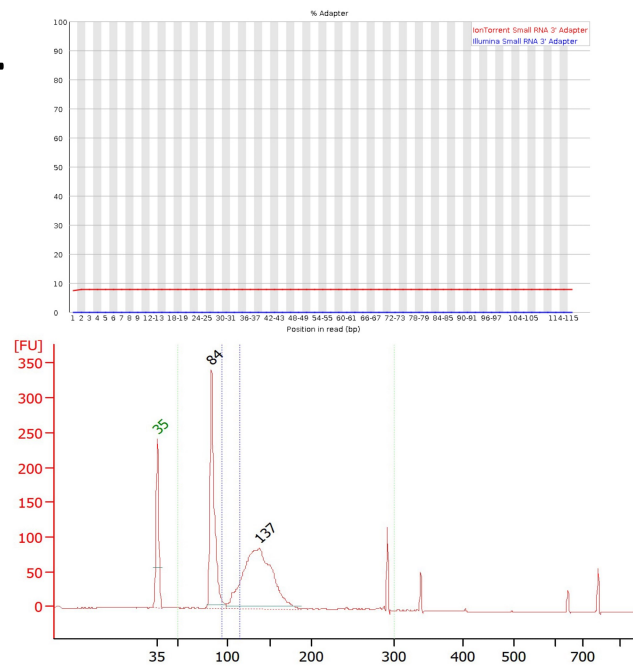

2

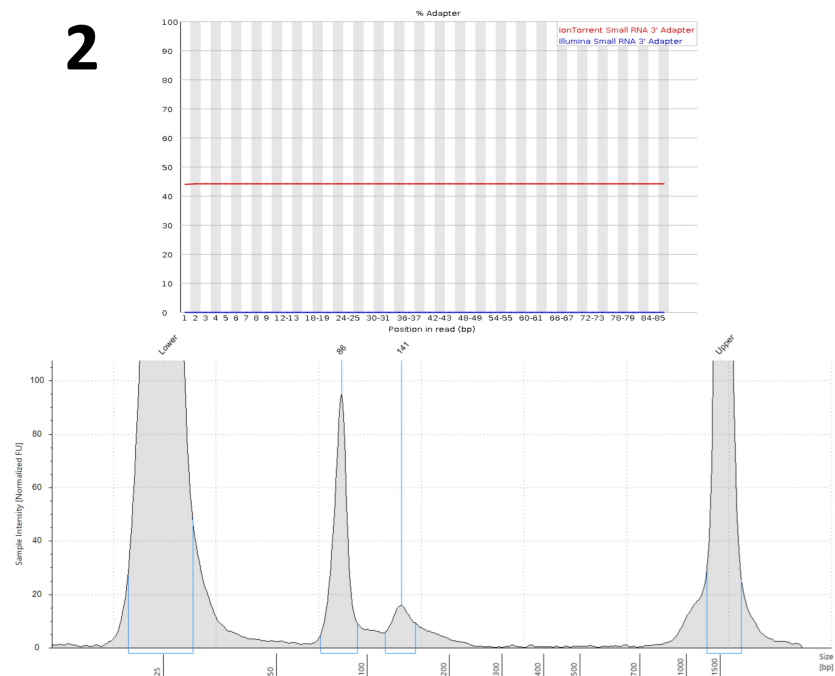

3

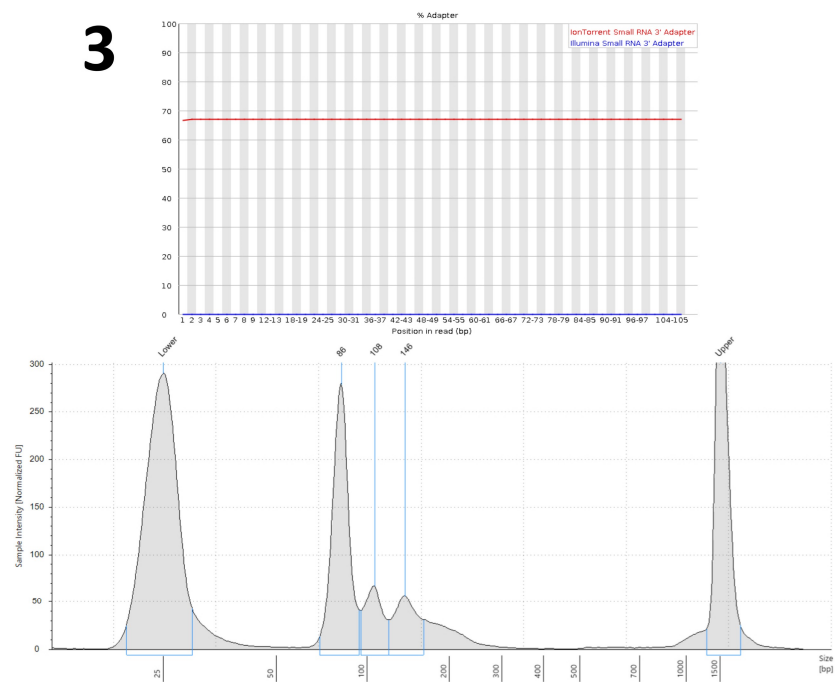

4

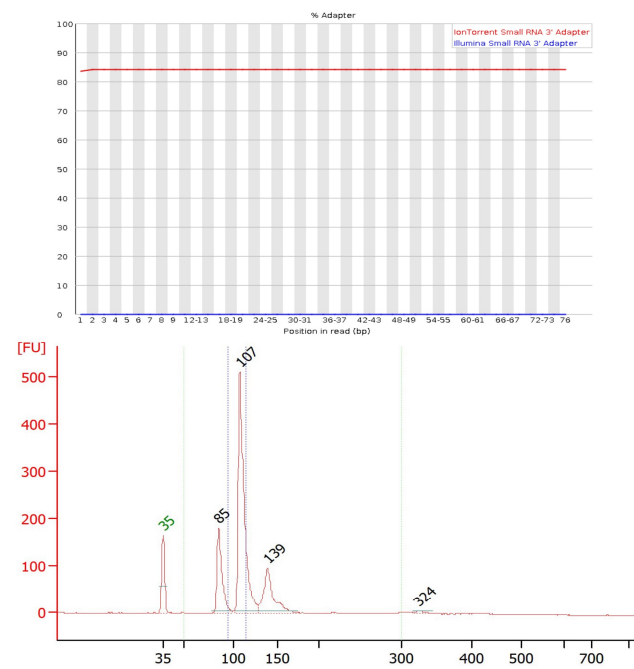

Supplement: Supplementary file 6 — Supporting Information [file JEX2-2-e91-s006.pdf]
