# Supplementary material for: Adapter dimer contamination in sRNA‐sequencing datasets predicts sequencing failure and batch effects and hampers extracellular vesicle‐sRNA analysis
Source: J Extracell Biol. 2023 Jun 11;2(6):e91. doi: 10.1002/jex2.91 (PMC11080836; doi:10.1002/jex2.91)
Supplement: Supplementary file 8 — Supporting Information [file JEX2-2-e91-s005.pdf]

Supplementary Table 1. Purified human milk EV

sample #	isolated sRNA (ng)	cDNA libraries (nM)	% read loss after pre-processing
1	73.1	3.7	31.9
2	57.2	3.1	14.6
3	217.1	6.7	9.8
4	132.1	5.9	8.4
5	72.2	4.0	14.1
6	194.0	5.7	18.1
7	209.2	2.9	7.9
8	55.3	3.8	15.1
9	194.0	6.0	6.7
10	173.5	5.1	9.4
11	240.0	4.5	10.6
12	45.1	3.8	40.5
13	35.4	4.4	28.4
14	143.3	5.3	7.3
15	50.5	2.9	20.6
16	63.4	3.4	5.3
17	231.1	3.6	20.9
18	81.7	4.4	11.6
19	127.8	4.8	14.0
20	72.1	4.4	14.9
21	60.8	5.7	30.6
22	89.8	2.3	13.6
23	29.0	7.1	37.8
24	86.2	2.2	19.6
25	63.5	2.4	23.1
26	237.5	3.3	14.0
27	128.9	2.7	10.2
28	36.4	2.0	24.7
29	316.1	6.7	24.0
30	58.0	3.1	19.3
31	10.7	2.2	36.3
32	84.4	2.2	34.4
33	172.1	4.2	38.6
34	32.5	5.6	63.8
35	60.5	2.7	46.8
36	71.0	5.1	58.8
37	69.0	6.3	59.1
38	69.7	8.4	71.6
39	110.3	8.4	78.4
40	95.9	8.9	86.6
