# Supplementary material for: Adapter dimer contamination in sRNA‐sequencing datasets predicts sequencing failure and batch effects and hampers extracellular vesicle‐sRNA analysis
Source: J Extracell Biol. 2023 Jun 11;2(6):e91. doi: 10.1002/jex2.91 (PMC11080836; doi:10.1002/jex2.91)
Supplement: Supplementary file 9 — Supporting Information [file JEX2-2-e91-s001.pdf]

Supplementary Table 2. EV-enriched human GC plasma

sample #	isolated miRNA (ng)	cDNA libraries (nM)	% read loss after pre-processing
1 (patient 1, BS)	1.1	0.3	54.2
2 (patient 4, AS2)	1.9	0.4	57.2
3 (patient 3, AS2)	3.1	0.2	59.2
4 (patient 4, BS)	2.8	3.2	80.4
5 (patient 4, AS1)	3.2	1.2	95.2
6 (patient 3, BS)	5.2	3.0	99.2
7 (patient 3, AS1)	2.7	7.1	99.7
8 (patient 2, AS2)	1.3	6.1	98.4
9 (patient 1, AS2)	0.9	0.4	98.7
10 (patient 1, AS1)	1.9	1.2	99.4
11 (patient 2, BS)	2.9	0.5	99.7
12 (patient 2, AS1)	4.4	1.4	99.9
