# Supplementary material for: Adapter dimer contamination in sRNA‐sequencing datasets predicts sequencing failure and batch effects and hampers extracellular vesicle‐sRNA analysis
Source: J Extracell Biol. 2023 Jun 11;2(6):e91. doi: 10.1002/jex2.91 (PMC11080836; doi:10.1002/jex2.91)
Supplement: Supplementary file 11 — Supporting Information [file JEX2-2-e91-s011.pdf]

Supplementary Table 4. Human GC FFPE tissues

sample #	isolated miRNA (ng)	cDNA libraries (nM)	% read loss after pre-processing
1	5.7	0.7	69.5
2	11.6	0.2	81.6
3	23.2	1.5	87.6
4	24.2	8.6	97.5
