# Supplementary material for: Adapter dimer contamination in sRNA‐sequencing datasets predicts sequencing failure and batch effects and hampers extracellular vesicle‐sRNA analysis
Source: J Extracell Biol. 2023 Jun 11;2(6):e91. doi: 10.1002/jex2.91 (PMC11080836; doi:10.1002/jex2.91)
Supplement: Supplementary file 12 — Supporting Information [file JEX2-2-e91-s012.pdf]

## *Supplementary Table 5. Purified human milk EV*

| sample # | % read loss | % short reads | % adapter dimers |
|---|---|---|---|
| 1 | 31.9 | 28.0 | 0.3 |
| 2 | 14.6 | 10.3 | 0.4 |
| 3 | 9.8 | 5.5 | 0.5 |
| 4 | 8.4 | 4.7 | 0.5 |
| 5 | 14.1 | 9.9 | 0.8 |
| 6 | 18.1 | 13.5 | 1.0 |
| 7 | 7.9 | 3.9 | 1.2 |
| 8 | 15.1 | 10.5 | 1.2 |
| 9 | 6.7 | 2.4 | 1.3 |
| 10 | 9.4 | 5.5 | 1.4 |
| 11 | 10.6 | 5.6 | 1.8 |
| 12 | 40.5 | 34.7 | 1.8 |
| 13 | 28.4 | 22.3 | 1.9 |
| 14 | 7.3 | 2.4 | 2.0 |
| 15 | 20.6 | 14.0 | 2.1 |
| 16 | 5.3 | 1.4 | 2.2 |
| 17 | 20.9 | 14.7 | 2.7 |
| 18 | 11.6 | 6.1 | 2.7 |
| 19 | 14.0 | 7.6 | 3.1 |
| 20 | 14.9 | 8.7 | 3.5 |
| 21 | 30.6 | 23.8 | 3.5 |
| 22 | 13.6 | 4.7 | 4.9 |
| 23 | 37.8 | 29.7 | 5.3 |
| 24 | 19.6 | 9.7 | 6.7 |
| 25 | 23.1 | 12.6 | 6.7 |
| 26 | 14.0 | 3.2 | 6.9 |
| 27 | 10.2 | 0.6 | 7.1 |
| 28 | 24.7 | 12.5 | 8.2 |
| 29 | 24.0 | 12.4 | 8.8 |
| 30 | 19.3 | 6.3 | 9.4 |
| 31 | 36.3 | 23.8 | 9.7 |
| 32 | 34.4 | 20.7 | 10.2 |
| 33 | 38.6 | 16.0 | 20.5 |
| 34 | 63.8 | 26.4 | 31.7 |
| 35 | 46.8 | 6.4 | 37.4 |
| 36 | 58.8 | 17.6 | 38.4 |
| 37 | 59.1 | 6.8 | 49.9 |
| 38 | 71.6 | 2.7 | 67.3 |
| 39 | 78.4 | 5.5 | 71.3 |
| 40 | 86.6 | 7.0 | 78.4 |
