# Supplementary material for: Adapter dimer contamination in sRNA‐sequencing datasets predicts sequencing failure and batch effects and hampers extracellular vesicle‐sRNA analysis
Source: J Extracell Biol. 2023 Jun 11;2(6):e91. doi: 10.1002/jex2.91 (PMC11080836; doi:10.1002/jex2.91)
Supplement: Supplementary file 13 — Supporting Information [file JEX2-2-e91-s015.pdf]

Supplementary Table 6. EV-enriched human GC plasma

sample #	% read loss	% short reads	% adapter dimers
1 (patient 1, BS)	54.2	20.8	28.9
2 (patient 4, AS2)	57.2	16.3	32.0
3 (patient 3, AS2)	59.2	12.9	35.4
4 (patient 4, BS)	80.4	7.4	69.4
5 (patient 4, AS1)	95.2	7.6	86.2
6 (patient 3, BS)	99.2	5.3	93.3
7 (patient 3, AS1)	99.7	4.5	94.6
8 (patient 2, AS2)	98.4	2.5	95.6
9 (patient 1, AS2)	98.7	0.4	97.6
10 (patient 1, AS1)	99.4	1.4	97.9
11 (patient 2, BS)	99.7	1.3	98.3
12 (patient 2, AS1)	99.9	1.1	98.7
