# Supplementary material for: Adapter dimer contamination in sRNA‐sequencing datasets predicts sequencing failure and batch effects and hampers extracellular vesicle‐sRNA analysis
Source: J Extracell Biol. 2023 Jun 11;2(6):e91. doi: 10.1002/jex2.91 (PMC11080836; doi:10.1002/jex2.91)
Supplement: Supplementary file 14 — Supporting Information [file JEX2-2-e91-s008.pdf]

Supplementary Table 7. EV-enriched mice plasma

sample #	% read loss	% short reads	% adapter dimers
1	85.9	46.6	36.9
2	87.3	42.0	42.4
3	79.6	33.9	42.7
4	80.3	34.4	42.9
5	83.6	33.4	47.5
6	87.0	35.9	48.5
7	88.1	37.0	48.7
8	86.5	33.7	50.2
9	84.2	27.1	54.2
10	86.0	27.9	55.6
11	86.9	26.9	57.3
12	92.6	29.3	60.7
13	91.8	27.5	61.7
14	92.1	26.9	62.7
15	92.3	23.5	65.8
16	88.8	14.0	71.7
