# Supplementary material for: Adapter dimer contamination in sRNA‐sequencing datasets predicts sequencing failure and batch effects and hampers extracellular vesicle‐sRNA analysis
Source: J Extracell Biol. 2023 Jun 11;2(6):e91. doi: 10.1002/jex2.91 (PMC11080836; doi:10.1002/jex2.91)
Supplement: Supplementary file 15 — Supporting Information [file JEX2-2-e91-s004.pdf]

Supplementary Table 8. Human GC FFPE tissues

sample #	% read loss	% short reads	% adapter dimers
1	69.5	43.7	15.8
2	81.6	27.1	51.9
3	87.6	12.8	72.9
4	97.5	5.2	91.6
